# Whole-exome sequencing in individuals with obsessive–compulsive disorder and chronic tic disorders identifies 36 large-effect risk genes

**DOI:** 10.1038/s41593-026-02419-5

**Published:** 2026-09-01

**Authors:** Belinda Wang, Matthew N. Tran, Sheng Wang, Yuting Liu, Emily Olfson, George Wang, Nawei Sun, Jeanselle Dea, Charles Ochieng’ Olwal, Lyvia Bertolace, Michael H. Bloch, Carolina Cappi, Yi-Chieh Chang, Denise Chavira, Barbara J. Coffey, Martha J. Falkenstein, Adam C. Frank, Martin E. Franklin, Stephanie Garayalde, Helena Garrido, Marco Grados, Rami Hatem, Allyna-London Howell, Starlette Khim, Jennie M. Kuckertz, Mindy M. Le, Allison Libby, Ryan J. McCarty, Mary E. McNamara, Daniel McNeil, Euripedes C. Miguel, Cara Nasello, Binh Nguyen, Tenzin Norbu, Lauren Oh, Ashley Ordway, Catherine Paciotti, Viviana A. Peskin, Christopher Pittenger, Helen Blair Simpson, Heather Simpson Martin, Max A. Tischfield, Jinchuan Xing, Jessica J. Zakrzewski, Tourette International Collaborative Genetics (TIC Genetics), Belinda Wang, Sheng Wang, Barbara J. Coffey, Cara Nasello, Max A. Tischfield, Jinchuan Xing, Juliane Ball, Noa Benaroya-Milshtein, Kate Bornais, Keun-Ah Cheon, Erik M. Elster, Dana Feldman, Carolin Fremer, Danea Glover, Tammy Hedderly, Isobel Heyman, Hyun Ju Hong, Chaim Huijser, Christina Kappler-Friedrichs, Heejoo Kim, Young Key Kim, Nadine Kirchen, Carolin Sophie Klages, Bennett L. Leventhal, Holan Liang, Maria Loreta Lopez, Osman Malik, Marieke Messchendorp, Dararat Mingbunjerdsuk, Pablo Mir, Astrid Morer, Kirsten R. Müller-Vahl, Alexander Münchau, Laura Muñoz-Delgado, Tara L. Murphy, Kerstin J. Plessen, Veit Roessner, Simon Schmitt, Chitra Shukla, Sara Sopena, Tamar Steinberg, Zsanett Tarnok, Joshua K. Thackray, Meitar Timmor, Anne Uhlmann, Ana Vigil-Pérez, Frank Visscher, Susanne Walitza, Andrea Dietrich, Donald L. Gilbert, Pieter J. Hoekstra, Young Shin Kim, Samuel Kuperman, Alyssa Rosen, Samuel H. Zinner, Robert A. King, Guy Rouleau, Jay A. Tischfield, Gary A. Heiman, Thomas V. Fernandez, A. Jeremy Willsey, Matthew W. State, Andrea Dietrich, Donald L. Gilbert, Pieter J. Hoekstra, Young Shin Kim, Samuel Kuperman, Alyssa Rosen, Samuel H. Zinner, Mehdi Bouhaddou, Robert A. King, Guy Rouleau, Kerry J. Ressler, Carol A. Mathews, Nevan J. Krogan, Nenad Sestan, Jay A. Tischfield, A. Moses Lee, Gary A. Heiman, Thomas V. Fernandez, A. Jeremy Willsey, Matthew W. State

**Affiliations:** 1 Department of Psychiatry and Behavioral Sciences, UCSF Weill Institute for Neurosciences, University of California, San Francisco, CA USA; 2 Department of Neuroscience, Yale School of Medicine, New Haven, CT USA; 3 Child Study Center, Yale University School of Medicine, New Haven, CT USA; 4 Department of Microbiology, Immunology, and Molecular Genetics, University of California Los Angeles, Los Angeles, CA USA; 5 Department of Psychiatry, University of Rochester, Rochester, NY USA; 6 Department of Psychiatry, Robert Wood Johnson Medical School, Rutgers, the State University of New Jersey, Piscataway, NJ USA; 7 Center for OCD, Anxiety and Related Disorders, University of Florida, Gainesville, FL USA; 8 Department of Psychology, University of California, Los Angeles, CA USA; 9 Department of Psychiatry and Behavioral Sciences, University of Miami Miller School of Medicine, Miami, FL USA; 10 OCD Institute, McLean Hospital, Belmont, MA USA; 11 Department of Psychiatry, Harvard Medical School, Boston, MA USA; 12 Department of Psychiatry and Behavioral Sciences, Keck School of Medicine, University of Southern California, Los Angeles, CA USA; 13 Rogers Behavioral Health, Philadelphia, PA USA; 14 Private practice, San Jose, Costa Rica; 15 Department of Psychiatry and Behavioral Sciences, Child and Adolescent Psychiatry, Johns Hopkins University, Baltimore, MD USA; 16 Department of Psychiatry and Human Behavior, Thomas Jefferson University Hospital, Philadelphia, PA USA; 17 Department of Psychiatry and Behavioral Sciences, Baylor College of Medicine, Houston, TX USA; 18 Departamento de Psiquiatria, Faculdade de Medicina, Universidade de São Paulo, Sao Paulo, Brazil; 19 Department of Genetics and the Human Genetics Institute of New Jersey, Rutgers, the State University of New Jersey, Piscataway, NJ USA; 20 Asociación de Psiquiatras de Argentina, Buenos Aires, Argentina; 21 Department of Psychiatry, Yale University School of Medicine, New Haven, CT USA; 22 Department of Neuroscience, Yale University School of Medicine, New Haven, CT USA; 23 Department of Psychology, Yale University Graduate School of Arts and Sciences, New Haven, CT USA; 24 Center for Brain and Mind Health, Yale University School of Medicine, New Haven, CT USA; 25 Wu-Tsai Institute, Yale University, New Haven, CT USA; 26 Department of Psychiatry, Columbia University, New York, NY USA; 27 Center for OCD and Related Disorders, New York State Psychiatric Institute, New York, NY USA; 28 UF Health Rehab Center, Norman Fixel Institute for Neurological Diseases, University of Florida, Gainesville, FL USA; 29 Department of Cell Biology and Neuroscience, Rutgers, the State University of New Jersey, Piscataway, NJ USA; 30 Child Health Institute of New Jersey, Robert Wood Johnson Medical School, New Brunswick, NJ USA; 31 Department of Psychiatry, University of California, San Diego, CA USA; 32 Department of Child and Adolescent Psychiatry, University Medical Center Groningen, University of Groningen, Groningen, The Netherlands; 33 Accare Child Study Center, Groningen, The Netherlands; 34 The Research School of Behavioral and Cognitive Neurosciences, University of Groningen, Groningen, The Netherlands; 35 Division of Neurology, Cincinnati Children’s Hospital Medical Center, Cincinnati, OH USA; 36 Department of Psychiatry and Behavioral Sciences, Dell Medical School, University of Texas at Austin, Austin, TX USA; 37 Department of Psychiatry, Carver College of Medicine, University of Iowa, Iowa City, IA USA; 38 Division of Neurology, Children’s Hospital of Philadelphia, Philadelphia, PA USA; 39 Department of Pediatrics, Division of Developmental Medicine, University of Washington School of Medicine, Seattle, WA USA; 40 Department of Neurology and Neurosurgery, Montreal Neurological Institute-Hospital, McGill University, Montreal, Quebec Canada; 41 Department of Psychiatry, University of Florida, Gainesville, FL USA; 42 Genetics Institute, University of Florida, Gainesville, FL USA; 43 Evelyn F. and William L. McKnight Brain Institute, University of Florida, Gainesville, FL USA; 44 Quantitative Biosciences Institute (QBI), University of California San Francisco, San Francisco, CA USA; 45 Department of Bioengineering and Therapeutic Sciences, University of California San Francisco, San Francisco, CA USA; 46 Gladstone Institute of Data Science and Biotechnology, J. David Gladstone Institutes, San Francisco, CA USA; 47 University Hospital of Psychiatry Zurich, Child and Adolescent Psychiatry and Psychotherapy, Zurich, Switzerland; 48 Department of Child Psychiatry, Feinberg Child Study Center, Schneider Children’s Medical Center of Israel, Petach Tikva, Israel; 49 Gray Faculty of Medical and Health Sciences, Tel Aviv University, Tel Aviv, Israel; 50 Department of Human Genetics, McGill University, Montreal, Québec Canada; 51 Yonsei University College of Medicine, Severance Hospital, Seoul, South Korea; 52 Department of Child and Adolescent Psychiatry, Faculty of Medicine of the TU Dresden, Dresden, Germany; 53 Clinic of Psychiatry, Social Psychiatry and Psychotherapy, Hannover Medical School, Hannover, Germany; 54 Evelina London Children’s Hospital, Guy’s and St Thomas’ NHS Foundation Trust, London, UK; 55 School of Life Sciences and Medicine, King’s College London, London, UK; 56 Great Ormond Street Hospital for Children NHS Foundation Trust, London, UK; 57 Great Ormond Street Institute of Child Health, University College London, London, UK; 58 Hallym University Sacred Heart Hospital, Anyang, South Korea; 59 Department of Child and Adolescent Psychiatry, Amsterdam UMC, Amsterdam, The Netherlands; 60 Academic Center for Child and Adolescent Psychiatry, Levvel, Amsterdam, The Netherlands; 61 Korea Institute for Children’s Social Development, Seoul, South Korea; 62 Department of Psychiatry, Yonsei Bom Clinic, Seoul, South Korea; 63 Irving B. Harris Professor of Child and Adolescent Psychiatry, Emeritus, The University of Chicago, Chicago, IL USA; 64 South London and Maudsley NHS Foundation Trust, London, UK; 65 Department of Neurology, Seattle Children’s Hospital, University of Washington School of Medicine, Seattle, WA USA; 66 Unidad de Trastornos del Movimiento, Servicio de Neurología, Instituto de Biomedicina de Sevilla (IBiS), Hospital Universitario Virgen del Rocío, CSIC, Universidad de Sevilla, Seville, Spain; 67 Centro de Investigación Biomédica en Red sobre Enfermedades Neurodegenerativas (CIBERNED), Instituto de Salud Carlos III, Madrid, Spain; 68 Departamento de Medicina, Facultad de Medicina, Universidad de Sevilla, Seville, Spain; 69 Department of Child and Adolescent Psychiatry and Psychology, Institute of Neurosciences, Hospital Clinic, Barcelona, Spain; 70 Institut d’Investigacions Biomèdiques August Pi i Sunyer (IDIBAPS), Barcelona, Spain; 71 Centro de Investigación Biomédica en Red de Salud Mental (CIBERSAM), Instituto Carlos III, Madrid, Spain; 72 University of Barcelona, Barcelona, Spain; 73 Institute of Systems Motor Science, Center of Brain, Behavior and Metabolism, University of Lübeck, Lubeck, Germany; 74 Child and Adolescent Mental Health Center, Mental Health Services, Capital Region of Denmark, Copenhagen, Denmark; 75 Division of Child and Adolescent Psychiatry, Department of Psychiatry, University Medical Center, University of Lausanne, Lausanne, Switzerland; 76 Department of Psychiatry, Psychotherapy and Psychosomatics, RWTH Aachen University, Aachen, Germany; 77 Division of Neonatology, Robert’s Center for Pediatric Research, Children’s Hospital of Philadelphia, Philadelphia, PA USA; 78 Vadaskert Child and Adolescent Psychiatric Hospital, Budapest, Hungary; 79 Egyenlito Diagnostic, Behavioral Therapy and Consultation Centre, Budapest, Hungary; 80 Department of Neurology, Admiraal De Ruyter Ziekenhuis, Goes, The Netherlands

**Keywords:** Next-generation sequencing, Genetic predisposition to disease, Rare variants, Rare variants, Psychiatric disorders

## Abstract

Obsessive–compulsive disorder (OCD) and chronic tic disorders (CTDs) are highly heritable. Rare mutations confer large risks for OCD and CTDs but only four high-confidence (hc) genes have been identified. Here we analyzed whole-exome sequencing data from 3,964 individuals with OCD, CTDs or both, including 2,418 trios. We found an excess in cases of de novo and rare protein-damaging mutations and identified 36 hc genes (false discovery rate < 0.1), including four previously identified hc genes (*CELSR3, CHD8, SCUBE1* and *WWC1*) and four genes that overlap with OCD genome-wide association study loci (*BRWD1*, *CELSR3*, *QRICH1* and *SYNE1*). Risk genes are shared among OCD, CTDs and other neurodevelopmental conditions. Transcriptomic and network analyses highlight mechanistic convergence and increased risk gene expression in postnatal cerebellum, prenatal and postnatal cortex and striatum. Dozens of large-effect OCD and CTD genes offer insights into pathogenesis and a path forward for illuminating pathophysiology and identifying novel treatment targets.

## Abstract (web summary)

The authors used whole-exome sequencing and analysis of rare coding variation in individuals with obsessive–compulsive disorder and chronic tic disorders, including Tourette disorder, to identify 36 large-effect risk genes, highlighting both shared genetic risks and convergent biology.

## Main

Obsessive–compulsive disorder (OCD) and chronic tic disorders (CTDs), including Tourette disorder (TD), are potentially disabling conditions that affect 1–2% of the population^1,2^. Both OCD and CTDs are highly heritable^3,4^; however, genetic studies to date have not yet substantially clarified pathogenesis and disease mechanisms or led to the identification of novel treatment targets.

OCD is characterized by intrusive thoughts or images (obsessions) and repetitive behaviors or mental acts (compulsions) performed to alleviate distress, typically beginning in late childhood or early adulthood^1^. CTD is defined by persistent recurrent motor movements and/or vocalizations, often preceded by premonitory urges, with typical onset in early childhood^2^. Both show a male predominance; however, in OCD, this has only been observed in childhood-onset cases^5^. Severe cases of OCD or CTD can result in life-long impairment and co-occurring neuropsychiatric conditions are common^1,6^.

OCD and CTD frequently co-occur. Specifically, 50% of individuals with CTDs exhibit obsessive–compulsive symptoms and 20–30% of those with OCD have a history of tics^6,7^. They share substantial heritability^8–10^ and neuroimaging, clinical and animal studies implicate dysfunction of the cortico-striato-thalamo-cortical (CSTC) circuitry^11,12^. These parallels suggest overlapping etiologies, although differing natural histories and treatment responses also point to possible disorder-specific pathogenic and pathophysiological mechanisms.

Given the heritability of OCD and CTD, efforts to define their genetic bases have been longstanding^13,14^. Similar to other prevalent medical and psychiatric conditions, common alleles of small effect explain a substantial proportion of the population risk for both conditions^4,15^. Recent genome-wide association studies (GWASs) identified 30 OCD loci from 53,660 cases^8^ but only a single CTD locus from 9,619 cases^16^. The identification of rare, pathogenic, large-effect coding variants offers a complementary strategy to gain insight into these disorders^17–22^. To date, however, only four genes identified by whole-exome sequencing (WES) reach well-accepted significance thresholds (false discovery rate (FDR) < 0.1) and are considered high-confidence (hc) markers for OCD (*CHD8* and *SCUBE1*)^17^ and CTD (*CELSR3* and *WWC1*)^20^.

Several decades of progress in identifying rare heterozygous mutations of large effect in other neurodevelopmental disorders (NDDs) has enabled studies of construct-valid animal models, revealed spatiotemporal and mechanistic convergence across risk genes^22^ and seeded clinical trials of mechanism-driven treatments^23^. Motivated by the potential for similar progress in OCD and CTD, we analyzed new and previously reported WES data from 3,964 probands affected with OCD, CTDs or both conditions—2,418 from parent–child trios and 1,546 singletons, roughly doubling prior case sample sizes^18,20^, alongside 1,734 control trios. The majority of case trios were simplex (neither parent affected; 88.2% OCD, 88.3% CTD and 84.9% both (OCDCTD)). Analysis of the disorders separately yielded 12 hcOCD and 10 hcCTD genes. Combining OCD and CTD probands yielded 34 OCDCTD hc genes. Two genes identified as hc in the disorder-specific analyses only met the threshold for a probable (p) risk gene (0.1 ≤ FDR < 0.3) in the combined cohort. All told, our analyses yielded 36 hc genes associated with one or both disorders.

Comparisons of OCD and CTD hc genes to other NDDs, including autism spectrum disorder (ASD)^24–28^, developmental delay and/or intellectual disability (DD/ID)^29^ and schizophrenia (SCZ)^30^, revealed both shared and distinct risks. This pattern was recapitulated in transcriptomic analyses, which showed elevated risk gene expression during prenatal cortical development, similar to ASD, as well as distinctively in later postnatal stages, consistent with the relatively later onset of OCD and CTDs. These analyses also find statistically significant increased expression in telencephalic projecting excitatory neurons. Lastly, analysis of gene interaction databases showed that OCD and CTD genes exhibit significantly increased connectivity. These findings, along with the results of both bulk brain and single-cell expression studies, point to spatiotemporally convergent biological processes and pathways underlying OCD and CTD.

## Results

### Cohort overview and study design

**Fig. 1:**
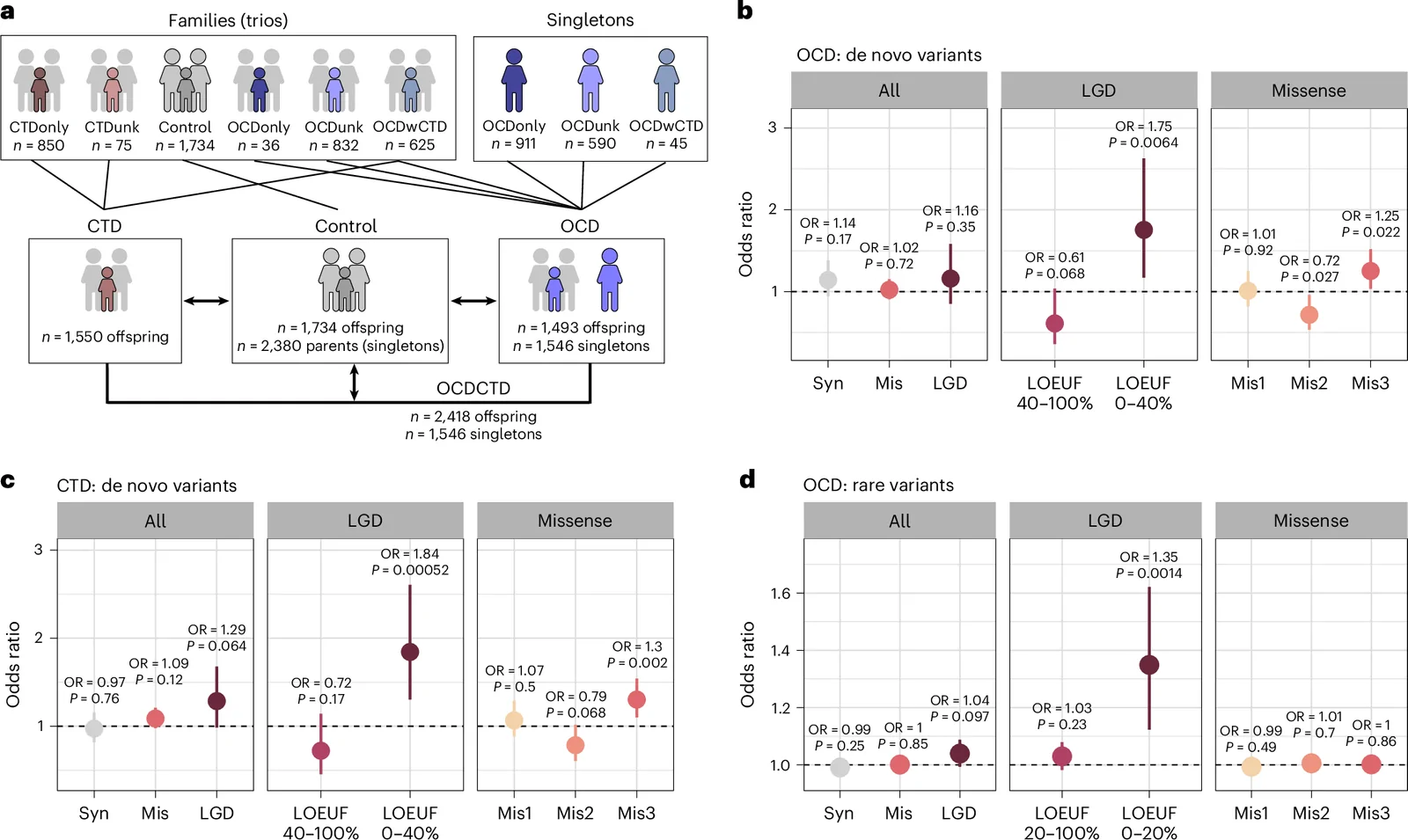
Gene discovery overview and dn and rare variant burden analysis. **a**, Cohort overview. Shown are the numbers of families (affected offspring with sequence data from both parents) and singletons (affected individuals without available parental data) contributing to trio-based and singleton analyses. Samples were classified by diagnostic status as CTDonly, CTDunk, OCDonly, OCDunk or OCDwCTD and grouped into OCD, CTD or combined OCDCTD cohorts for gene discovery analyses. Most case families were simplex (neither parent affected; 88.2% OCD, 88.3% CTD and 84.9% OCDCTD). **b**–**d**, Case-versus-control odds for dn variants in OCD trios (**b**), dn variants in CTD trios (**c**) and rare variants in OCD singletons (**d**). Allele frequency (AF) thresholds in a population ref. ^34^ were ≤0.05% for trio dn variants and ≤0.005% for singleton rare variants. Results are shown for all variants (left; Syn, missense and LGD), LGD variants stratified by genic constraint (middle; lower LOEUF deciles indicate higher constraint) and missense variants classified by predicted deleteriousness (right; Mis3 = most deleterious^71^). Odds ratios (ORs) (points) and 95% confidence intervals (error bars) from two-sided logistic regression of case–control status on per-individual variant count are shown; case and control sample sizes are as in **a**. Consistent with established convention^17,20,21,26,30,35^, *P* values are not corrected for multiple tests. Source data

We analyzed new and previously sequenced WES data from individuals with OCD, CTDs or both, as well as neurotypical controls (Fig. 1a, Supplementary Note 1 and Supplementary Tables 1–3). After quality control, the case cohort included 2,418 parent–proband trios and 1,546 singletons. Controls included 1,734 trios of neurotypical siblings and their parents from the Simons Simplex Collection (SSC)^31^; parents of European ancestry were used as singleton controls (*n* = 2,380). In total, 872 trios (36%) and 1,080 singletons (70%) were newly sequenced for this study.

Trio probands reflected a range of diagnostic subtypes, including OCD without CTD (OCDonly; *n* = 36), OCD with co-occurring CTD (OCDwCTD; *n* = 625), OCD with unknown CTD status (OCDunk; *n* = 832), CTD without OCD (CTDonly; *n* = 850) and CTD with unknown OCD status (CTDunk; *n* = 75) (Fig. 1a and Supplementary Table 2). All case singletons carried an OCD diagnosis (OCDonly, *n* = 911; OCDunk, *n* = 590; OCDwCTD, *n* = 45; Supplementary Table 3).

All WES data were processed through a unified pipeline with standardized alignment and variant calling (Methods and Supplementary Fig. 1). Quality control confirmed reported family structures and excluded samples that failed sequencing quality metrics or showed unexpected relatedness (Supplementary Figs. 2 and 3 and Supplementary Tables 4 and 5). All singleton analyses were restricted to individuals of European ancestry. To reduce batch effects, analyses were restricted to coding regions meeting stringent quality metrics across all sources (Methods and Supplementary Table 1).

Building on prior OCD^17,18^, CTD^20,21^ and NDD^25,26,29,30^ studies, we evaluated three classes of rare variation (Methods): (1) rare de novo (dn) mutations in families (allele frequency (AF) ≤ 0.05% in a population reference database^32^); (2) rare transmitted variants in families (AF ≤ 0.005%); and (3) rare variants in singletons (AF ≤ 0.005%). Dn versus transmitted status could not be distinguished in the singleton cohorts and, thus, all variants are referred to as rare. In trio probands, we identified 4,028 dn variants (Supplementary Table 6) and 308,078 transmitted variants. In singletons, we identified 296,736 rare variants.

We categorized variants by predicted functional impact: (1) likely gene-disrupting (LGD, including frameshift, stop-gain and essential splice site changes) and (2) missense variants predicted to be the most damaging (Mis3)^17,18,21,33^. LGD variants were further stratified by gene-level ‘loss of function (LoF) observed/expected upper bound fraction (LOEUF)’ scores^34^. We set specific threshold criteria for constraint in the current study on the basis of our empirical data (Supplementary Note 2). We refer to the combined set of LGD and Mis3 variants as damaging (Dam) variants. Synonymous (Syn) variants were used as a control.

### Rare Dam variants contribute to OCD and CTD risk

We first compared variant frequencies between cases and controls using two complementary analytic approaches: logistic regression to assess whether individuals carrying at least one variant were more likely to be cases (Fig. 1b–d and Supplementary Fig. 4d) and Poisson or negative binomial regression to evaluate whether cases carried more variants overall (Supplementary Fig. 4a–c,e and Methods). Reported *P* values are unadjusted, following the convention established in prior WES studies^17,20,21,26,30,35^.

Across all cohorts (OCD, CTD and OCDCTD), cases showed elevated rates of dnLGD in LoF-intolerant genes, specifically among the lowest four LOEUF deciles. DnMis3 variants were also elevated in cases versus controls, consistent with prior WES studies^17,18,20,21^ (Fig. 1b,c and Supplementary Fig. 4). We observed no differences in transmitted LGD or transmitted Mis3 variants in trios, even within the lowest LOEUF decile (Supplementary Table 7b). OCD singletons showed a higher frequency of rare LGD variants in the two lowest LOEUF deciles, in line with previous work^18^ (Fig. 1d and Supplementary Fig. 4). Syn variants did not differ between cases and controls (Fig. 1b–d and Supplementary Fig. 4). In sum, given these results, we included only dnLGD, dnMis3 and rare LGD variants in gene discovery analyses (Supplementary Note 2, Supplementary Figs. 5–7 and Supplementary Tables 7, 8 and 10).

**Fig. 2:**
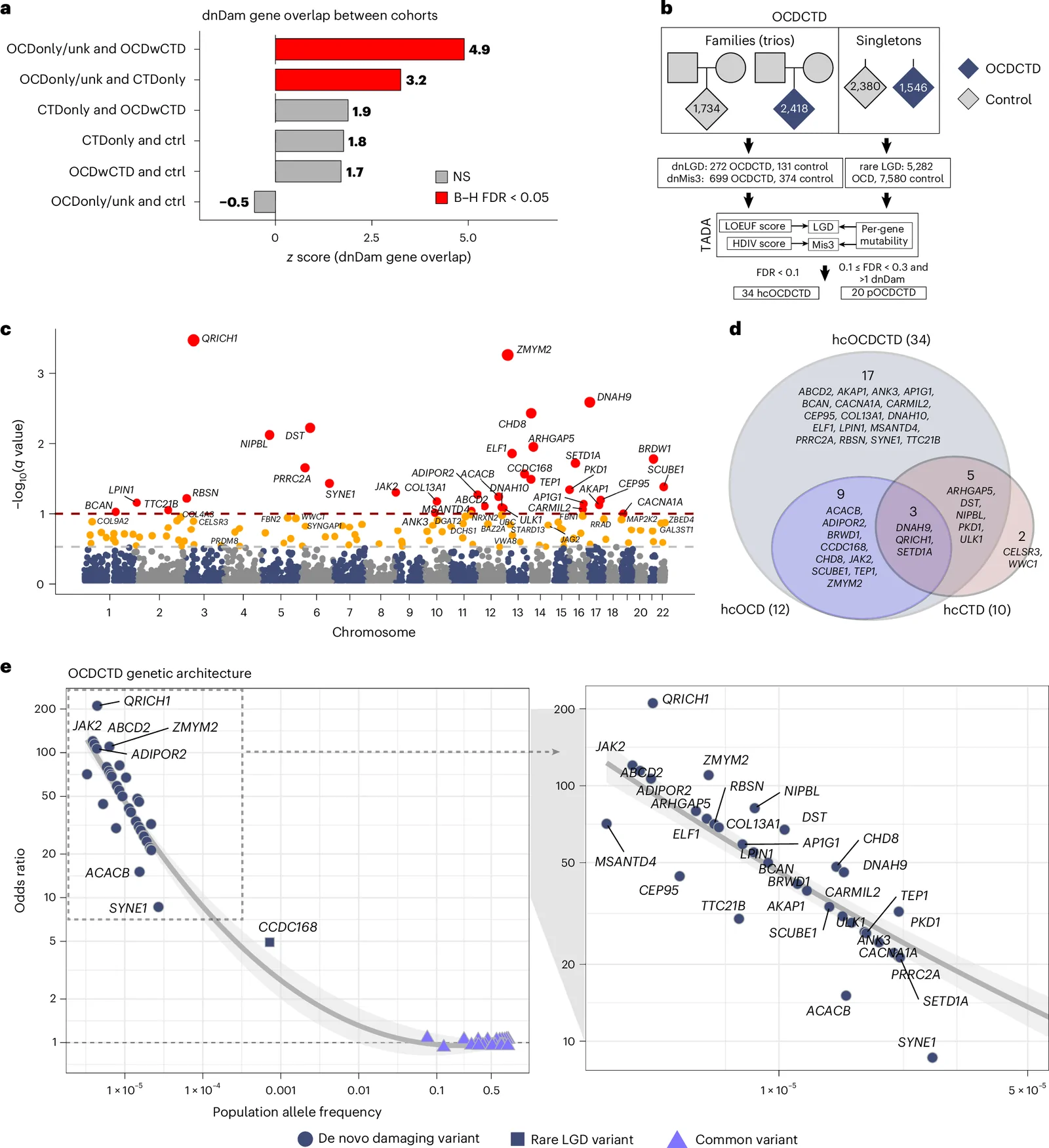
Genetic association results for OCD, CTD and OCDCTD. **a**, Overlap of genes with dnDam variants between cohort pairs to assess shared genetic risk between OCD and CTD. Cohorts include offspring with CTDonly, OCDwCTD, OCDonly/unk and neurotypical controls. One-sided *z* scores indicate the extent to which the observed gene overlap exceeds chance on the basis of 10,000 random permutations. Bars are colored by Benjamini–Hochberg-corrected FDR < 0.05 (red). NS, not significant (gray). **b**, Sequencing data for the OCDCTD cohort were analyzed using a Bayesian framework (TADA) that incorporated predicted deleteriousness of LGD and missense variants and gene-specific mutability. Total variants observed per cohort are shown. Genes with FDR < 0.1 were defined as hc; those with 0.1 ≤ FDR < 0.3 and >1 dnDam variant were defined as p. **c**, Manhattan plot of −log_10_(*q*) by chromosomal position for the OCDCTD cohort. Dashed lines denote FDR thresholds of 0.1 and 0.3. Points are colored by FDR (red, <0.1; orange, ≥0.1 and <0.3), with labels shown for hc and p genes only. **d**, Overlap among hcOCD, hcCTD and hcOCDCTD risk genes. Numbers indicate gene counts. **e**, Population AF versus OR for hcOCDCTD risk genes and OCD GWAS loci^8^. Shapes indicate variant class and the line shows the LOESS fit of the OR across population AF, with the shaded band representing the 95% confidence interval. Source data

Various lines of genetic evidence have long suggested shared risk between OCD and CTD^17,36^. Consequently, we assessed overlap in genes carrying dnDam variants across three trio cohorts of similar size: OCDwCTD, CTDonly and OCDonly/unk (OCDonly and OCDunk were combined because of the small size of OCDonly). Permutation testing revealed significantly greater overlap than expected by chance between CTDonly and OCDonly/unk and between OCDonly/unk and OCDwCTD (Fig. 2a and Supplementary Table 9). No significant overlap was observed with controls.

CTD has previously been shown to have a strong male sex bias^2^. Male predominance in OCD has been identified only in childhood-onset cases^1,5^. Among our trio probands, we observe a male-to-female ratio of 3.8:1 for CTD and 1.4:1 for OCD. A prior study reported elevated dnLGD rates in LoF-intolerant genes among males with OCD^18^. Using Poisson regression, we observe no sex-specific differences in dn variant rates in any variant class (Methods, Supplementary Fig. 8 and Supplementary Tables 11 and 12). We further examined relationships between dn variant status and additional clinical phenotypes, observing no differences by comorbidity (OCDwCTD versus OCDonly/unk or CTDonly) or by age at onset (Methods and Supplementary Fig. 8). However, these genotype–phenotype analyses were constrained by incomplete phenotypic data and small subgroup sample sizes.

Using maximum-likelihood estimation (MLE), we estimated that 550 OCD, 1,094 CTD and 760 OCDCTD genes contribute to risk through dn and rare variants. These results were robust to alternative analytic methods (Methods and Supplementary Table 13). On the basis of the observed excess frequency in cases versus controls, we estimated that 19–50% of dnDam, and rare LGD variants likely contribute to OCD and/or CTD (Supplementary Table 14). At the individual level, we estimated that 7–8% of OCD and/or CTD probands carry at least one risk-contributing dnDam variant and 3.0% of OCD singletons carry a rare LGD variant (Supplementary Table 15).

### Identification of large-effect risk genes

We applied a well-established Bayesian framework, transmission and dn association (TADA)^26,37^, to integrate evidence from dnLGD, dnMis3 and rare LGD variants (Methods, Fig. 2b and Supplementary Fig. 9). For each autosomal protein-coding gene, we calculated a Bayes factor (BF) summarizing the cumulative evidence for association, accounting for gene-specific mutation rates and the estimated number of contributing risk genes. Consistent with prior studies^17,20,21,25,38^, genes with FDR < 0.1 were designated hc and those with 0.1 ≤ FDR < 0.3 were classified as p. However, given the identification of many p genes on the basis of only a single dnDam mutation, we elected to restrict the p designation further to those genes meeting the p FDR threshold and carrying at least two dnDam variants (Supplementary Tables 16 and 17).

Using these definitions, the OCD cohort alone identified 12 hcOCD and 16 pOCD genes, while the CTD cohort alone yielded ten hcCTD and six pCTD genes. An omnibus analysis of both cohorts together identified 34 hcOCDCTD and 20 pOCDCTD genes (Fig. 2c,d, Supplementary Fig. 9 and Supplementary Tables 16 and 17). In total, 36 genes met the hc criteria in at least one analysis (OCD, CTD or OCDCTD). Two hcCTD genes, *CELSR3* and *WWC1*, no longer met the hc threshold in the combined OCDCTD analysis. However, both genes remained hc in an analysis of OCDCTD restricted to trios. Four hcOCDCTD genes (*ABCD2*, *DNAH9*, *DNAH10* and *TEP1*) showed higher rates of rare LGD mutations in singleton controls versus cases; however, three of these (*ABCD2, DNAH9* and *DNAH10*) were hc both in the omnibus analysis and among OCDCTD trios. *TEP1* was a p gene in the trio-only OCDCTD analysis and hc in the combined OCDCTD analysis (Supplementary Table 16 and Supplementary Note 3).

Given that the current study includes previously reported samples, we did not aim to independently replicate published results. However, our findings do not alter the designation of the previously identified hcOCD genes *CHD8* and *SCUBE1* (ref. ^17^) or the hcCTD genes *CELSR3* and *WWC1* (refs. ^20,21^). Moreover, in our study, an additional dnMis3 variant was present in *WWC1* in a newly identified OCDwCTD proband^18^. In contrast, several previously reported genes were no longer identified as hc or p (Supplementary Note 4). This includes *SLITRK5*, which previously approached genome-wide significance in an OCD case–control analysis. Of note, the prior association was supported by transmitted and singleton rare Mis3 variants. We did not rely on these classes of variation for gene discovery in the current study given the absence of significantly elevated rates in our cases versus controls (Fig. 1d, Supplementary Fig. 4c and Supplementary Table 7b).

Collectively, we found hc genes identified herein carrying very large effects, with odds ratios (ORs) indicating up to 210-fold increases in risk (57-fold on average; Fig. 2e, Supplementary Fig. 10 and Supplementary Table 18). In the current study, the gene *CCDC168* was identified solely through rare LGDs and confers intermediate risk (OR = 4.9), consistent with the expectation that rare transmitted LGD variants will be found to carry effect sizes falling between dnDam variants and common alleles.

**Fig. 3:**
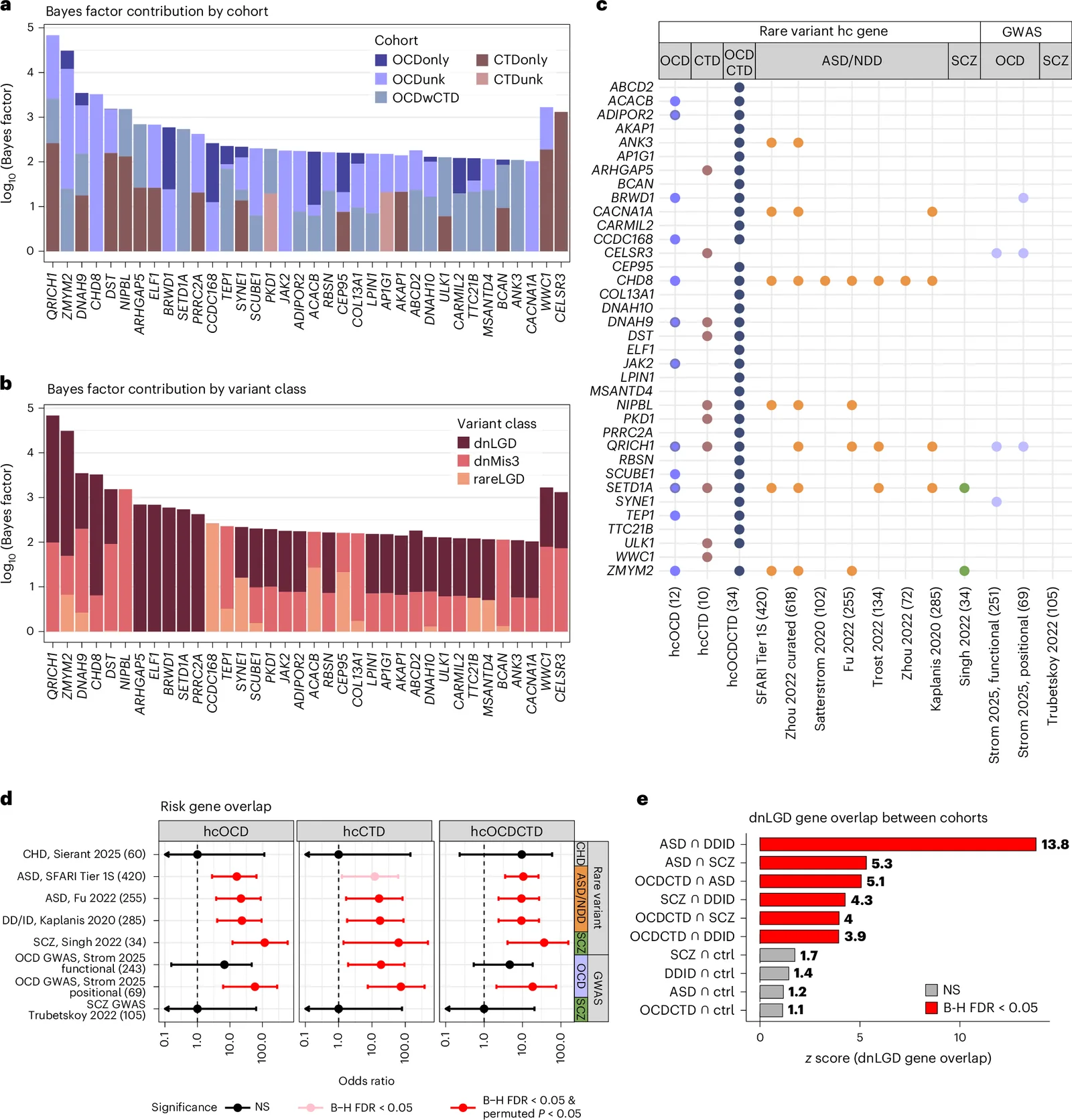
Risk gene profiles and overlap with other NDDs. **a**,**b**, Contribution of phenotype (**a**) and variant class (**b**) to the statistical evidence (quantified by BF^37^) for each of the 36 hc (FDR < 0.1) risk genes identified in OCD, CTD or OCDCTD. **c**, Overlap of the hc genes identified in this study with previously published rare-variant-associated gene sets for ASD, NDDs and SCZ and genes that map to OCD and SCZ GWAS loci^8,24–30,72^ (Methods). Colored dots indicate gene set membership; publications are given by first author and year and gene set sizes are in parentheses. **d**, Enrichment of hcOCD, hcCTD and hcOCDCTD genes in previously reported neuropsychiatric gene sets. ORs (points) and 95% confidence intervals (error bars) are shown from two-sided Fisher’s exact tests comparing hcOCD (*n* = 12), hcCTD (*n* = 10) and hcOCDCTD (*n* = 34) genes to external gene sets, including ASD/NDD, SCZ, CHD and OCD GWAS loci^8,24–30,40^. Gene set sizes are in parentheses. The unit of analysis was individual genes. Colors: NS, black; Benjamini–Hochberg FDR < 0.05, pink; Benjamini–Hochberg FDR < 0.05 and empirical *P* < 0.05 (10,000 permutations), red. ORs for sets with zero overlap were set to 1 for visualization. **e**, Overlap of genes with dnLGD variants across OCDCTD, ASD, DD/ID, SCZ and neurotypical controls^26,29,30^. One-sided *z* scores indicate the extent to which the observed gene overlap exceeds chance on the basis of 10,000 random permutations. Bar colors: Benjamini–Hochberg FDR < 0.05, red; NS, gray. Source data

To explore how the various phenotypes contributed to gene discovery, we decomposed BFs to determine which cohort subgroups contributed to the identification of each of the 36 hc genes (Methods). In total, 30 of 36 (83.3%) genes had evidence for association derived from both OCD and CTD cases (Fig. 3a, Supplementary Fig. 11 and Supplementary Table 19). Only one gene (*CELSR3*) was supported exclusively by mutations in CTDonly. Five genes (*BRWD1*, *CACNA1A*, *CCDC168*, *CHD8* and *JAK2*) were supported only by variants in OCD cases without CTD (OCDonly and OCDunk). We also assessed the relative contributions of various variant classes and found that dnLGD and dnMis3 mutations from trios contributed more evidence for gene discovery than rare LGD variants in singleton cases (Fig. 3b, Supplementary Fig. 11 and Supplementary Table 19). Several genes were supported solely by dnLGD mutations (*ARHGAP5*, *BRWD1*, *ELF1*, *PRRC2A* and *SETD1A*), one gene was supported only by dn missense mutations (*NIPBL*) and, as noted, one gene was supported only by rare LGD variants in singletons (*CCDC168*).

### Shared genetic risk for OCD and CTD derives from both common and rare variants

The largest OCD GWAS meta-analysis to date identified 30 genome-wide significant loci, with 31 additional loci found to be significant in subgroup analyses^8^. We compiled two risk gene sets—one based on functional or regulatory connections to the 30 genome-wide significant loci (‘functional’) and one based on physical proximity to the 61 loci (‘physical’)—and compared these to the 36 hc genes identified in the present study. Four hc genes (*BRWD1, CELSR3*, *QRICH1* and *SYNE1*) were present in both the rare variant and the common allele gene sets, exceeding chance expectations (Fig. 3c,d and Supplementary Table 20). In line with this, gene-level multimarker analysis of genomic annotation (MAGMA)^39^, which tests whether aggregated single-nucleotide polymorphism (SNP) associations implicate a gene, showed nominal enrichment for seven hc genes, including *CELSR3* and *QRICH1* (Supplementary Fig. 12 and Supplementary Note 5). While *CELSR3* showed significant association only with CTD in the current study, the overlap observed with genome-wide significant OCD GWAS results suggests that it is likely to be associated with both phenotypes.

### Shared genetic risk with NDDs

Given previous findings in other NDDs^24–30^, we reasoned that the rare variants identified in the current study might contribute to a broad range of diagnoses. We tested two complementary hypotheses that (1) genes carrying at least one dnLGD mutation (termed dnLGD genes) in the current study would overlap with those in ASD, SCZ and/or DD/ID cohorts and (2) hcOCD and hcCTD risk genes would overlap with established ASD, DD/ID and SCZ risk genes.

We confirmed both hypotheses. dnLGD genes in the current study significantly overlapped with dnLGD genes in ASD, DD/ID and/or SCZ (*z* scores of 3.9–5.1 by permutation testing; Fig. 3e, Supplementary Fig. 17 and Supplementary Table 21). Moreover, hcOCD and hcCTD genes significantly overlapped with established ASD, DD/ID and SCZ risk genes identified in prior WES studies and in curated databases^24–30^ (Fig. 3c,d and Supplementary Table 20) but not with established congenital heart disease (CHD) genes^40^ (*P* = 1).

We next evaluated gene-level FDR values for all the identified hc genes with respect to their association with OCDCTD and compared these to results from recent large-scale WES studies of ASD^26^, DD/ID^26,29^ and SCZ^30^ (Supplementary Note 6, Supplementary Fig. 14 and Supplementary Table 22). These analyses suggest that it may be possible in the future to differentiate a subset of OCDCTD-predominant or OCDCTD-specific genetic risks.

### Spatial, temporal and cellular mapping of risk genes

We evaluated the spatial, temporal and cellular expression patterns of OCD and CTD risk genes using multiple transcriptomic atlases^41–47^. To query high-resolution microarray and bulk RNA-seq datasets, we relied on smaller and more stringently defined sets of risk genes, namely hc and p genes. Alternatively, to increase the power of exploratory analyses, we relied on a larger set of genes carrying at least one dnLGD mutation to query sparser single-cell RNA-seq datasets.

**Fig. 4:**
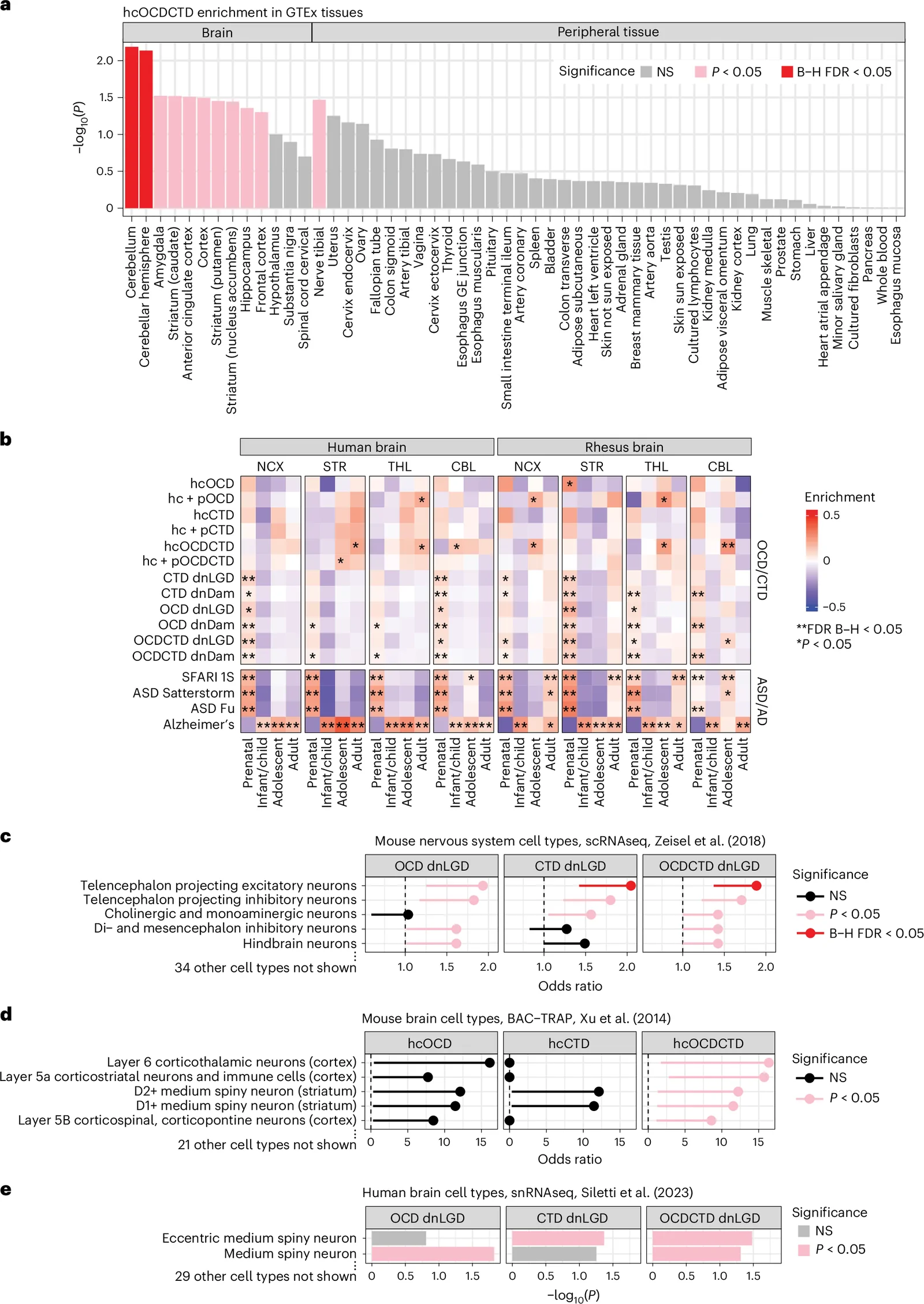
Regional, developmental and cell type risk gene enrichment. **a**, Enrichment of hcOCDCTD genes across 54 GTEx human tissues^41^. **b**, Developmental expression of hc, p, dnLGD (genes with ≥1 observed dnLGD) and dnDam (genes with ≥1 observed dnDam) gene sets in human^43^ and rhesus macaque^44^ neocortex (NCX), striatum (STR), thalamus (THL) and cerebellum (CBL). Heat maps show enrichment relative to all other genes (one-sided Wilcoxon rank-sum test). HcASD^25,26^ gene sets were included as a control for prenatal-biased expression and an Alzheimer’s disease (AD)^49^ gene set as a control for postnatal-biased expression. **Benjamini–Hochberg FDR < 0.05; *nominal *P* < 0.05. **c**–**e**, Risk gene set enrichment among the top 10% cell-type-specific genes from 39 mouse postnatal nervous system cell types^45^ (**c**), mouse postnatal brain cell-type-specific markers defined by BAC–TRAP^46,73^ (**d**) and 31 adult human brain cell types^47^ (**e**). To balance specificity and power, smaller hc gene sets were queried in **d** (higher-resolution microarray data) and larger dnLGD sets were queried in **c**,**e** (sparser single-cell data). Only cell types with significant enrichment in at least one gene set are shown. Color coding (**a**,**c**–**e**): red, Benjamini–Hochberg FDR < 0.05; pink, nominal *P* < 0.05; gray, NS. Enrichment was calculated by gene set enrichment analyses using expression specificity ranks (**a**,**e**) or one-sided Fisher’s exact tests with ORs and 95% confidence intervals shown (**c**,**d**). Source data

Using GTEx bulk RNA-seq data from 54 human tissues^41^, we ranked genes by tissue specificity^48^ and performed ranked gene set enrichment analysis (Methods). Expression in the cerebellum was significantly increased after correcting for multiple testing. In addition, nominal enrichment was observed in the cortex (frontal and anterior cingulate), striatum (caudate, putamen and nucleus accumbens), amygdala and peripheral nervous system (tibial nerve) (Fig. 4a and Supplementary Table 23). No enrichment was detected in 40 non-nervous system tissues. The findings in cerebellum were not previously reported from OCD and CTD GWASs^8,16^.

We next evaluated spatial and temporal expression of OCD and CTD risk genes using the BrainSpan human brain atlas^42,43^, as well as a rhesus macaque atlas^44^ as a primate comparator. We focused on the neocortex, striatum, thalamus and cerebellum across prenatal to adult stages; regions were selected on the basis of the identification of adequate sample sizes. For each region, we computed gene-level temporal specificity *z* scores and used one-sided Wilcoxon rank-sum tests to assess whether risk genes showed greater stage-specific expression than background genes (Methods). ASD^24–26^ and Alzheimer’s disease (AD)^49^ were used as controls. HcOCDCTD genes showed elevated postnatal expression in the cerebellum (Fig. 4b and Supplementary Fig. 15). Across regions, dnLGD and dnDam genes in OCD, CTD and OCDCTD showed prenatal enrichment, with several regions and time points remaining significant after multiple-testing correction (FDR < 0.05). Hc and p risk genes showed nominally elevated postnatal expression, particularly in the human striatum and thalamus, a pattern distinct from ASD (Fig. 4b, Supplementary Fig. 15 and Supplementary Table 24).

We also mapped risk genes to cell-type-specific markers from postnatal transcriptomic atlases. Using a mouse nervous system single-cell RNA-seq atlas^45^, we tested whether dnLGD genes were overrepresented among the 10% most cell-type-specific genes^50^ using one-sided Fisher’s exact tests. Across 39 cell types, telencephalic projecting excitatory neurons, which include various subclasses (layer 2–6 cortical pyramidal neurons, hippocampal CA1/subicular neurons and basolateral amygdalar neurons), were significantly enriched after multiple-testing correction (Fig. 4c, Supplementary Fig. 16 and Supplementary Table 25). Nominal enrichment was also observed in telencephalic projecting inhibitory neurons (mainly dorsal and ventral striatal D1+ and D2+ medium spiny neurons), hindbrain, diencephalic/mesencephalic inhibitory and cholinergic/monoaminergic neurons. We observed concordant nominal enrichment in layer 5b and layer 6 cortical pyramidal neurons and striatal D1+ and D2+ medium spiny neurons with hc genes and an orthogonal mouse bacterial artificial chromosome-translating ribosome affinity purification (BAC–TRAP) dataset, which profiled ribosome-bound transcripts across 26 genetically defined nervous system cell populations by microarray^46^ (Fig. 4d, Supplementary Fig. 16 and Supplementary Table 26). These results are also consistent with prior OCD and CTD GWAS-based analyses^8,16,45^, which found that common variants were most enriched in telencephalic projecting excitatory and inhibitory neurons for OCD and in telencephalic projecting excitatory and diencephalic/mesencephalic inhibitory neurons for CTD.

Lastly, we evaluated a human adult brain single-nucleus RNA-seq atlas that profiled over 3 million nuclei and identified 31 cell types^47^. Genes were ranked by cell type specificity, defined as the proportion of each gene’s expression attributable to a given cell type. We applied ranked gene set enrichment analysis to assess whether genes carrying dnLGD mutations were overrepresented among the most cell-type-specific genes and find nominal enrichment in two medium spiny neuron subtypes (Fig. 4e, Supplementary Fig. 16 and Supplementary Table 27).

### Functional connectivity of OCD and CTD risk genes

We conducted a Gene Ontology (GO) analysis^51^, which found significant overrepresentation of ‘axonogenesis’ (GO:0007409) and ‘extracellular matrix structural constituent’ (GO:0005201) in the combined set of hcOCDCTD and pOCDCTD genes (Supplementary Fig. 17 and Supplementary Table 28).

**Fig. 5:**
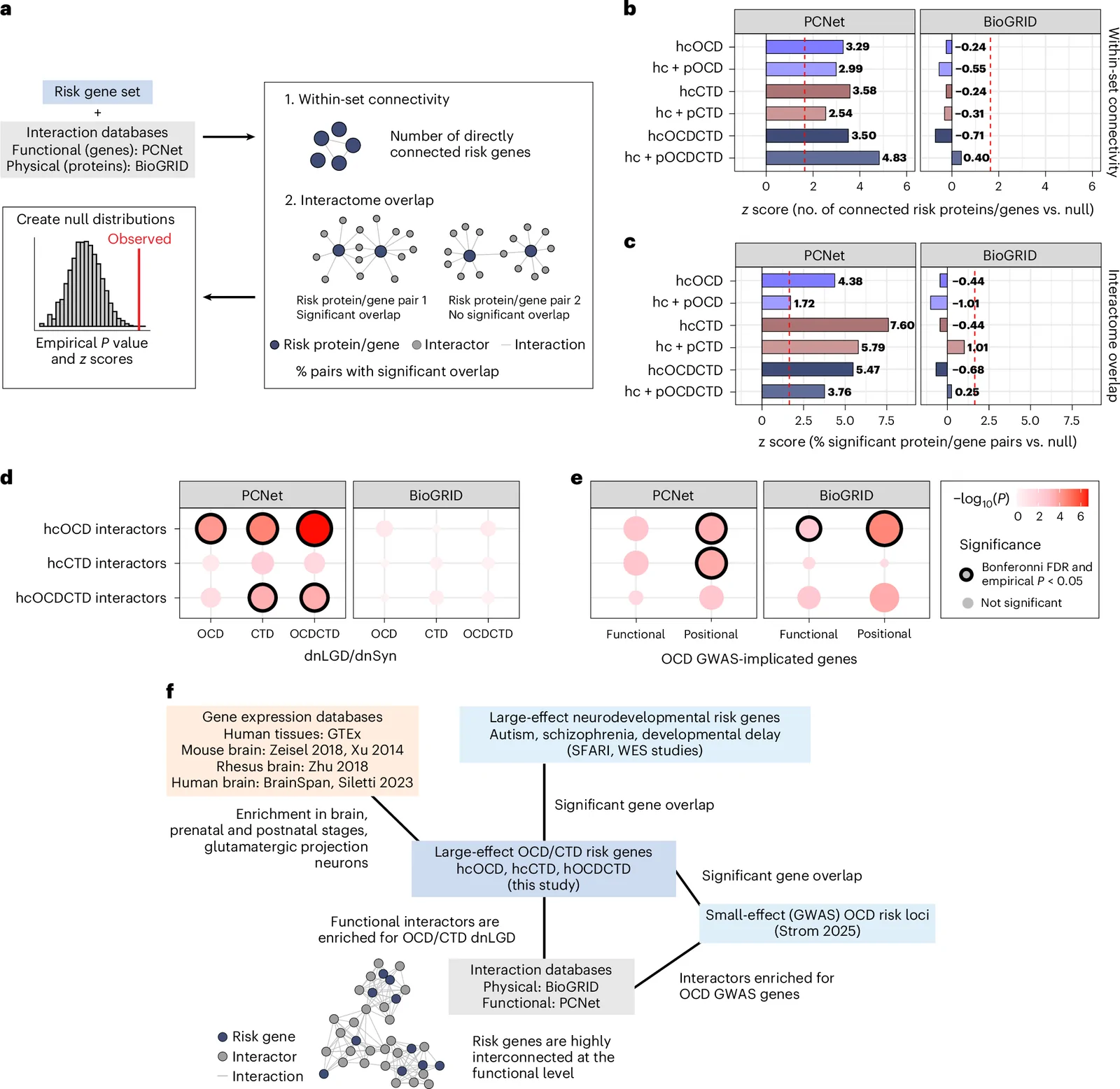
Network analysis reveals functional convergence of OCD and CTD genetic risk. **a**, Framework for assessing connectivity among risk proteins and genes in two interaction networks: PCNet (multimodal functional gene interactions, including PPIs) and BioGRID (physical PPIs)^52,56^. Within each network, we quantified (1) within-set connectivity, defined as the number of directly interacting risk proteins or genes, and (2) interactome overlap, defined as the percentage of risk protein–gene pairs with significant overlap among their first-degree interactors (one-sided Fisher’s exact test, *P* < 0.05, not adjusted for multiple comparisons). **b**,**c**, The *z* scores for within-set connectivity (**b**) and interactome overlap (**c**) across networks and various risk protein and gene sets (color-coded). Significance was assessed using permutation tests based on 10,000 (**b**) or 1,000 (**c**) null permutations. The *z* scores and empirical *P* values were derived from the resulting null distributions. Red lines indicate the nominal one-sided threshold for *P* < 0.05 (*z* > 1.64), not adjusted for multiple comparisons. **d**,**e**, Enrichment among the first-degree (direct) interactors of hc and hc + p risk proteins and genes in PCNet and BioGRID for dnLGD versus dnSyn variants observed in OCD, CTD or OCDCTD trio probands (**d**) and genes mapped to OCD GWAS loci (**e**)^8^. Dot size and color indicate –log_10_(*P*) from two-sided Fisher’s exact tests; black outlines denote significance (Bonferroni-adjusted *P* < 0.05 and permutation-derived empirical *P* < 0.05). **f**, Summary of genetic overlap, tissue and cell type specificity and functional convergence findings. Source data

We next assessed risk gene network connectivity using PCNet^52^, which integrates multiple types of functional interactions, including protein interactions, coexpression and regulatory relationships. For each hc and p gene set, we measured (1) within-set connectivity, defined as the number of risk genes that directly interact with at least one other risk gene in the set, and (2) interactome overlap, defined as the proportion of risk gene pairs sharing more interactors than expected by chance, among all possible risk gene pairs (Methods and Fig. 5a). The results were compared to null distributions from random gene sets to calculate *z* scores, where *z* > 1.64 corresponds to one-sided *P* < 0.05. We found significantly elevated within-set connectivity for hcOCD (8/12, *P* = 0.0038), hcCTD (7/10, *P* = 0.0021) and hcOCDCTD (24/34, *P* = 0.0001) (Fig. 5b, Supplementary Fig. 18a,b and Supplementary Table 29). The interactome overlap results followed a similar pattern, with significant overlap for all three gene sets (all *P* < 0.001; Fig. 5c, Supplementary Fig. 18c and Supplementary Table 30).

We examined whether first-degree interactors were enriched for pathogenic mutations (Methods, Fig. 5d,e and Supplementary Tables 31 and 32). HcOCD and hcOCDCTD interactors were significantly enriched for case dnLGD variants (Fisher’s exact test, Bonferroni-adjusted *P* < 0.01; empirical *P* < 0.01). Interactors of hcOCD and hcCTD also overlapped significantly with genes mapped to OCD GWAS loci^8^ (Fisher’s exact test, Bonferroni-adjusted *P* < 0.005; empirical *P* < 0.05). MAGMA gene set-level analysis showed nominal enrichment of OCD common variant risk among PCNet interactors of hcOCD, hcCTD and hcOCDCTD (Supplementary Fig. 18d and Supplementary Table 33).

We then explored the functional roles of hc genes using network propagation, which distributes signals from ‘seed’ genes across the PCNet interaction network to quantify connectivity with other genes. Analyses were performed separately with hcOCD, hcCTD and the union of hcOCD, hcCTD and hcOCDCTD as seed genes. GO overrepresentation analysis with the top 100 genes most significantly connected to the seed sets revealed enrichment of Hippo signaling and ‘establishment or maintenance of cell polarity’ for hcCTD, consistent with our prior CTD WES study^20^, telomere-related and DNA biosynthesis-related terms for hcOCD and ‘microtubule-based movement’ and cilia-related terms across all hc seed sets, consistent with our prior work on ASD risk genes^53,54^, including the hcOCDCTD gene *CHD8* (ref. ^55^) (Supplementary Fig. 19 and Supplementary Table 34).

We performed similar analyses using BioGRID^56^, which catalogs only physical protein–protein interactions (PPIs). Using this dataset, we did not replicate our findings (Fig. 5a–e, Supplementary Fig. 19 and Supplementary Tables 29–33). However, we note that this relatively sparse resource was likely underpowered for the present analyses and recent empirical protein interaction studies point to substantial limitations in the currently available public PPI databases, including BioGRID^57^. In contrast, the consistency of findings across multiple different approaches in the PCNet-based analyses suggests that OCD and CTD risk genes form functionally coherent networks, with their direct interactors enriched for both rare and common variant risk.

## Discussion

The present study identifies 36 hc genes from separate and combined studies of OCD and CTD cohorts, 34 of which meet hc thresholds in an omnibus OCDCTD analysis. We found that dn and rare pathogenic mutations are carried by approximately 3–8% of affected individuals with an average OR of 57. These findings markedly expand the number of known genes contributing to OCD and CTDs and the identification of rare coding mutations and large-effect risk genes lays a strong foundation for further study of construct-valid genetic models^58,59^, PPIs^33,57^ and spatiotemporal convergence of genetic risks^33,57,60–62^.

Our results confirm substantial genetic overlap between OCD and CTD. Notably, we found significant overlap in rare and common variant risk, with four hc genes overlapping with OCD GWAS loci^8^. These findings point to the potential for shared pathobiology between rare and common forms of these disorders, which, in turn, raise the prospect of overlapping mechanistically informed treatment approaches. The findings also underscore the importance of future studies investigating the full spectrum of genetic variation. In this context, the present analyses focus on rare coding single-nucleotide variants (SNVs) and small indels and do not include other classes of rare variation, such as copy-number variants (CNVs), noncoding variants or mitochondrial DNA. A comparison of our findings with previously reported OCD and CTD CNVs^20,63–65^ is detailed in Supplementary Note 7.

We found that OCDCTD risk genes overlap with ASD, DD/ID and SCZ but not CHD. Notably, however, some OCDCTD risk genes showed no association with ASD or DD/ID, despite the hundreds of large-effect hc genes already identified for these NDDs^26,29^. Although further gene discovery will be necessary to power definitive analyses, these results suggest that there may be both shared and distinctive genetic contributions to OCDCTD. Preliminary transcriptomic analyses support this view, showing similar patterns of expression enrichment in the prenatal brain in OCDCTD and ASD, along with evidence for distinctive postnatal enrichment in OCDCTD that is largely absent in our ASD analyses.

We found OCD and CTD risk genes are highly expressed in the cortex, striatum and thalamus during both prenatal and postnatal development and the cerebellum during postnatal development. Future studies will be aided both by expanded developmental expression datasets and additional gene discovery in OCD and CTDs. Notably, the most stringently defined analyses presented here, focused on hc genes, showed elevated postnatal brain expression, while the broader set of genes identified by a dnLGD mutation showed prenatal enrichment similar to that observed with hcASD. These differences likely reflect both biological factors and power limitations, as hc sets are smaller and enriched for higher-penetrance genes. Supporting the idea that risk spans both prenatal and postnatal stages, both the hc genes and the broader set of genes showed elevated expression in similar cell types. These include postnatal cortical excitatory projection neurons and striatal medium spiny neurons, consistent with the CSTC circuit implicated in OCD and CTD through neuroimaging, neuromodulation, animal models and common variant studies^8,66–70^. Our network propagation analysis highlights several mechanistic themes, including cell polarity, microtubule and cilia biology, that align with prior CTD findings^20^ and have also emerged prominently in our work in ASD^53–55^.

Although we found multiple lines of evidence demonstrating increased connectivity and functional convergence among OCDCTD risk genes, a commonly used PPI database, BioGRID^56^, did not replicate these findings. This discrepancy may reflect the fact that PCNet captures functional relationships relevant for OCDCTD that extend beyond direct PPIs. Alternatively, the absence of a signal may simply reflect inherent limitations of publicly available PPI resources^57^. The empirical study of the full set of proteins encoded by the 36 hc risk genes and their interactions, along with an assessment of the impact of pathogenic mutations, promises to deepen our understanding of mechanistic convergence, disorder pathogenesis and potential treatment targets^33,57^.

Overall, our results introduce a tenfold increase in the number of bona fide large-effect risk genes contributing to obsessions, compulsions and tics, help to clarify the genetic architecture of OCD and CTD, confirm and extend key neurobiological hypotheses and offer promising avenues to leverage large-effect coding mutations and resulting phenotypic overlap and divergence to elaborate pathophysiology.

## Methods

### Study participants

All participants (or parents, with minor assent) provided written consent under site-specific Institutional Review Board-approved protocols. Where provided, participant compensation consisted of a gift card up to 50 USD. Sex and, where available, age of onset are reported in Supplementary Table 2. WES data were aggregated from prior OCD^17,18^ and CTD^20,21^ studies and new TIC Genetics^74^ and Foundation for OCD Research (FFOR) samples. Controls were SSC^75–77^ neurotypical siblings (trio controls) and parents (singleton controls). The dataset before quality control (QC) comprised 15,716 samples (10,261 cases, 5,455 controls). Ascertainment criteria and before and after QC counts by source are detailed in Supplementary Note 1 and Supplementary Tables 1–3.

### Sample processing and variant identification

Supplementary Fig. 1 provides an overview of sample and variant QC.

#### Sequencing and alignment

New OCD and CTD exomes were captured with the Twist Bioscience Comprehensive Exome Panel (TwistBio) and sequenced on Illumina NovaSeq 6000 (S4 flowcells, 100-bp paired-end reads, ~100× mean coverage). Capture and metadata for all samples are in Supplementary Tables 2–3. Data processing followed GATK Best Practices^78^ using GATK (v.4.2.0) and Picard (v.2.25.0), including BWA-MEM (v.0.7.15-r1140) alignment to GRCh38 (SamToFastq > MergedBamAlignment), MarkDuplicatesSpark, BaseQualityScoreRecalibration and contamination estimation with VerifyBamID2 (v.1.0.6; FREEMIX, divided by 0.75 to correct underestimation). Per-sample gVCFs were generated with HaplotypeCaller.

#### Joint genotyping, hard filters and variant annotation

Samples were combined with GenomicsDB, joint-genotyped with GnarlyGenotyper and filtered by VQSR (pass at 99.7% truth-set sensitivity for SNVs and indels). Using Hail (v.0.2), we applied hard filters (DP ≥ 10, GQ ≥ 20), removed low-complexity regions^79^ and excluded ChrY variants in females and non-PAR ChrX variants in males. Variants were annotated using Ensembl VEP (v.113)^80^ and GENCODE (v.47) with gnomAD (v.4.1) allele frequencies and gene-level constraint (LOEUF, pLI)^32,34^, PolyPhen-2_HDIV (v.2.2.3)^71^ and MPC (v.2) scores^81^ (based on gnomAD v.2.1.1).

#### Consensus and callable regions

To limit platform, coverage and capture bias, we defined ‘consensus callable’ regions. For trios, GRCh38 coding regions were intersected with targets from seven capture platforms (±100 bp of padding), retaining sites with DP ≥ 15 in all family members. For singletons, coding regions were intersected with four platforms covering >95% of samples, retaining sites with DP ≥ 10 and GQ ≥ 20 in ≥90% of samples across the three most common platforms (each representing ≥10% of cases). These consensus callable regions were used for all burden analyses. For gene discovery, dn variants were included from a broader ‘restricted callable’ region (padded targets, DP ≥ 15 in all trio members) to maximize sensitivity, consistent with previous studies^20,21^.

#### Sample-level QC

Sample-level QC involved four steps. First, Hail identity-by-descent confirmed parent–child relationships (IBD_HAT ≥ 0.35; Z1 ≥ 0.7) and identified duplicate samples (IBD_HAT ≥ 0.95), which were resolved by prioritizing complete trios. Second, exome capture integrity was assessed by principal component analysis (PCA) with genome-wide coverage across 25,000 intervals^82^. Samples with unmatched capture profiles or with discordant trio clustering were excluded. Third, sex was inferred with Hail impute_sex; trios lacking the expected parental pattern were removed. Ancestry was inferred by PCA and a random forest classifier trained on 1000 Genomes superpopulations (scikit-learn v.1.3.1). EUR individuals (probability ≥ 0.8) were retained for singleton analyses (Supplementary Fig. 3 and Supplementary Table 7). Fourth, for trios, dn outliers (>99.7th percentile in Poisson distribution) were removed, leaving 4,136 high-quality trios. Gene discovery analyses also included previously Sanger-validated dn variants from 16 trios excluded by current QC, yielding 4,152 trios in total (2,418 cases and 1,734 controls; Supplementary Table 4). For singletons, we analyzed VQSR pass variants with ≥90% coverage across three main exome kits and removed outliers by call rate (3 s.d. below the mean), singleton count (3 s.d. above the mean), and het/hom and Ts/Tv ratios (3 s.d. above or below the mean), leaving 3,926 EUR singletons (1,546 cases and 2,380 controls; Supplementary Table 5).

#### dn variants

Candidates were heterozygous in probands and homozygous reference in both parents, with cohort AF ≤ 0.001, DP ≥ 15 in all members and proband AB 0.3–0.7. Variants were scored with DeNovoCNN^83^ and filtered to AF ≤ 0.0005 across all gnomAD (v.4.1) exome populations (global, African/African American, admixed American, Ashkenazi Jewish, East Asian, Finnish, Middle Eastern, non-Finnish European, South Asian and unassigned). Candidates without prior Sanger validation were manually reviewed with Integrative Genomics Viewer (https://igv.org). Confirmation rates (indels: 93.75%; SNVs: 99.65%) were consistent with prior reports^18,84^. Previously Sanger-validated variants^17,18,20,21^ from samples failing current QC were retained for gene discovery but excluded from burden testing (20 dn from 16 samples). Two previously failed variants were excluded. We retained one variant per gene per family, prioritizing the most Dam allele. Post-QC dn variants are in Supplementary Table 6.

#### Transmitted rare variants

Variants met DP ≥ 10, GQ ≥ 20, AB 0.3–0.7, VQSR pass and rarity (internal AF ≤ 0.0005 across 4,136 trios; global gnomAD AF ≤ 0.00005) thresholds. To mitigate platform-specific bias, SNV VQSLOD thresholds were calibrated using Hail’s transmission_disequilibrium_test on singleton Syn SNVs (AC = 1 in parents) separately in control (Roche NimbleGen EZ Exome v2) and case (TwistBio, the predominant case capture) trios. Thresholds were selected from the VQSLOD percentile bin showing the most balanced transmission (binomial test against 50% transmission, highest *P* value)^25,85^, yielding cutoffs of VQSLOD ≥ −1.444 (OCD/CTD) and VQSLOD ≥ −2.177 (SSC). Indels were filtered at VQSLOD ≥ −1.812 (99.5% tranche). Burden analyses included transmitted (proband GT 0/1, parent GT 0/1) and untransmitted (proband GT 0/0, parent GT 0/1) variants, requiring DP ≥ 15 and rarity thresholds (AF ≤ 0.00005 for non-Finnish Europeans; AF ≤ 0.0005 for other populations). For each gene, the most Dam allele per individual was retained.

#### Singleton rare variants

For QC-passing EUR samples, we retained heterozygous genotypes (GQ ≥ 20, DP ≥ 10, allele balance 0.25–0.75) passing the same VQSLOD thresholds as transmitted rare variants, with MAC ≤ 5 in the combined cohort and gnomAD global and non-Finnish-European AF ≤ 0.00005. For each gene, the most Dam allele per individual was retained.

### Burden analyses

#### Variant classification

We classified dn (trios), transmitted (trios) and singleton rare variants as Syn, missense or LGD (stop-gain, frameshift and splice donor/acceptor). We stratified LGD variants by LOEUF^85^ and missense variants by PolyPhen-2 HDIV^71^ (Mis3: probably Dam, Mis2: possibly Dam, and Mis1: benign) to enrich for deleterious variants. Syn variants were used as a negative control to confirm model calibration (Supplementary Figs. 5–7 and Supplementary Tables 7–10). Detailed rationale for the selected variant classifiers is provided in Supplementary Note 2.

#### Burden testing

To evaluate the contribution of rare coding variants to disease risk, we assessed variant burden between cases and controls (Fig. 1, Supplementary Fig. 4 and Supplementary Tables 7 and 8). We used logistic and Poisson regression for dn variants and logistic and negative binomial regression for transmitted variants. These complementary models evaluated both carrier frequency and per-individual burden. Analyses were performed for OCD, CTD and combined OCDCTD cohorts; rare variant analyses were restricted to the OCD cohort because of limited CTD singleton samples. To mitigate batch effects, all analyses were restricted to hc consensus callable coding regions. Statistical models were implemented in R, with 95% confidence intervals calculated as the estimate ± 1.96 s.e. The model specifications were as follows:

- Dn, logistic: case status ~ variant count + consensus callable base pairs
- Dn, Poisson: variant count ~ case status + offset(log(consensus callable base pairs))
- Transmitted, logistic: case status ~ transmitted variant count + untransmitted variant count + sex + ancestry PC1–PC10
- Transmitted, negative binomial: transmitted variant count ~ case status + untransmitted variant count + sex + ancestry PC1–PC10
- Rare, logistic: case status ~ variant count + sex + ancestry PC1–PC10
- Rare, negative binomial: variant count ~ case status + sex + ancestry PC1–PC10

We also assessed the potential influence of sex and clinical features (age of onset, comorbidity) on dn burden (Supplementary Fig. 8 and Supplementary Table 12). Using Poisson regression, we compared dn variant rates between male cases and controls, female cases and controls and male and female cases, as well as across clinical subgroups (comorbidity status). Associations with age at onset were evaluated within cohorts using two-sided Wilcoxon rank-sum tests; for OCDCTD, age at onset was defined as the earlier of the two disorders. All analyses were restricted to samples with available phenotypic data.

#### Contribution to risk

To quantify the proportion of disease risk attributable to rare variants, we estimated the variant rate ratio (RR = case rate/control rate) using Poisson (dn) or negative binomial (rare) regression with previously described covariates. The fraction of variants contributing to risk was estimated as 1 − 1/RR, with 95% confidence intervals derived from log-transformed RR bounds (Supplementary Table 14). To estimate the proportion of probands carrying ≥1 risk variant, we calculated the case–control difference in carrier frequency (using dnDam variants for trios; rare LGDs in genes with LOEUF 0–20% for singletons) and assessed significance and 95% confidence intervals by bootstrapping (1,000 replicates; Supplementary Table 15).

### Cross-disorder overlap of genes affected by dnDam variants

We tested whether genes affected by dnDam mutations were shared more than chance between OCD and CTD subgroups using family-based subgroups of comparable size (OCDwCTD, CTDonly and OCDonly/unk) and neurotypical SSC siblings as a negative control; OCDonly and OCDunk combined because of the small cohort size of OCDonly). We compared observed cohort overlaps against 10,000 size-matched mutability-weighted null permutations, calculated one-sided empirical *P* values and *z* scores and corrected for multiple testing using the Benjamini–Hochberg method (Fig. 2a and Supplementary Table 9)

### Gene discovery

#### Number of risk genes

The number of risk genes contributing to CTD, OCD and OCDCTD was estimated using MLE^20,21,86^ and unseen species^21,87^ frameworks, including all dnDam variants in the restricted callable interval. MLE used 10,000 permutations for 1–3,000 risk gene counts to find which best fit the observed distribution of genes with 1, 2 or ≥3 dnDam mutations. The ‘unseen species’ framework estimates risk gene count (*C*) as follows:

$$C=\frac{c}{u}+1\times d\times \frac{1-u}{u}$$

where *d* is the number of risk-carrying variants (observed minus expected dnDam, with expected values scaled from SSC controls), *c* is the number of observed risk genes (*d* − recurrent variants + genes with recurrent variants), *c*_1_ is the number of genes mutated once and *u* is the probability that a variant hits a previously mutated gene (1 − *c*_1_*/d*). Confidence intervals were obtained by recalculating *C* using Poisson-derived bounds for expected control dnDam counts^17^. Both methods gave similar estimates (Supplementary Table 13) and MLE values were used in TADA^17,20,21^.

#### TADA framework for gene discovery

Risk genes were identified using the TADA framework^37^, a Bayesian model that integrates evidence across variant classes (dn and rare), considers control variants and allows flexible inclusion of gene-level constraint metrics and functional annotations^26^ (https://github.com/talkowski-lab/TADA_2022). TADA computes a BF for each gene and variant class based on observed variant counts, cohort sizes, estimated number of underlying risk genes, and gene-specific and variant class-specific prior probabilities. For primary gene discovery, we included variant classes that showed significant enrichment in burden analyses (OCD and OCDCTD: dnLGD (stratified by LOEUF 0–40%), dnMis3 and rare LGD (LOEUF 0–20%); CTD: dnLGD (stratified by LOEUF 0–40%) and dnMis3). We considered 18,111 coding genes with gnomAD constraint metrics. TADA parameters included sample size (number of trios for dn, singletons for rare variants), π (fraction of causal genes = MLE estimate/18,705 consensus genes), μ (gene-level mutability, estimated by gnomAD4.1 constraint; LGD: lof.mu, Mis3: mis.mu scaled by the SSC control proportion of Mis3/all missense), λ (case/control fold enrichment from Poisson or negative binomial regression) and γ (relative risk, calculated as γ = 1 + (λ − 1)/π, floored at 1 to exclude protective estimates). Full parameter values are in Supplementary Table 16. Gene-level BFs were multiplied across variant classes and converted to posterior probabilities to estimate FDR *q* values. All BF values (conservatively including <1, evidence against association) were retained (Supplementary Note 3). We defined hc (FDR < 0.1) and p (0.1 ≤ FDR < 0.3) risk genes using established statistical thresholds^17,20,21,38^. We further required p genes to carry ≥2 dnDam variants because evidence driven by a single variant is sensitive to gene-specific mutability estimates. TADA was implemented for OCD, CTD and OCDCTD, with trio-only and trio-plus-singleton analyses for OCD and OCDCTD (Supplementary Tables 16 and 17); hc and p gene counts are detailed in Supplementary Table 17.

### Risk gene characteristics

#### Risk gene architecture

Each gene received one relative risk estimate per cohort from case-versus-control rates, prioritizing dn > rare > common variant evidence. For dn variants, relative risk was the observed dnDam count in probands divided by the expected dn count from gene-specific gnomAD (v.4.1) mutation rates (TADA μ); for rare variants, it was the ratio of allele frequencies in OCD singletons (*n* = 1,546) versus gnomAD (v.4.1) exomes (*n* = 730,947) and, for common variants, it was the ORs and allele frequencies reported for 30 genome-wide significant OCD GWAS loci^8^. A LOESS trend with 95% confidence interval summarized relative risk across the population AF spectrum (Fig. 2e, Supplementary Fig. 10 and Supplementary Table 18).

#### Variant class and cohort contributions to risk gene associations

To determine the contribution of specific variant classes and phenotypes to association evidence for the 36 hc genes, we decomposed TADA evidence (BFs) by variant class and proband phenotype. Variant class BFs were extracted from TADA. Phenotype-level BFs were computed by scaling each log_10_(BF) by the share of variants from probands with a given phenotype: scaled log_10_(BF) = log_10_(max(1, BF)) × *n* phenotype/Σ *n*. BFs < 1 were floored at 1 for visualization (affecting *ABCD2*, *CELSR3* and *WWC1*; Supplementary Note 3). Scaled phenotype scores were summed across variant classes to yield total support per diagnostic group (Fig. 3a,b and Supplementary Table 19).

### Risk gene overlap

#### Overlap with external risk genes

We compared hcOCD, hcCTD and hcOCDCTD with rare variant risk gene sets from ASD, DD/ID, NDD, SCZ and CHD WES studies and with OCD and SCZ GWAS gene sets. Rare variant sets were as follows: SFARI Tier 1S (tier 1 + syndromic, release January 13, 2025 from SFARI Gene^24^), Zhou (2022)^28^ curated dominant/X-linked ASD/NDD, ASD TADA FDR < 0.1 from Satterstrom (2020)^25^, Fu (2022)^26^ and Trost (2022)^27^, Zhou (2022) (SPARK) study-wide-significant genes, Kaplanis (2020)^29^ exome-wide-significant DD/ID genes and Singh (2022)^30^ SCZ (SCHEMA) meta-analysis *q* < 0.1. GWAS sets were Strom (2025) functional (243 of original 251 HGNC-mapped genes linked to 30 lead SNPs through at least one of six mapping approaches^8^), Strom (2025) positional (69 genes within ±50 kb of 61 genome-wide significant lead SNPs in the main GWAS or subanalyses of the four ascertainment subgroups, extracted from Strom 2025 Supplementary Table 3) and Trubetskoy (2022)^72^ (genes associated with 287 lead SCZ SNPs through fine mapping that had matching HGNC symbols). Pairwise overlap significance was calculated using two-sided Fisher’s exact tests with background as the intersection of genes analyzed in both studies and Benjamini–Hochberg correction. Empirical significance was calculated using 10,000 permutations with sampling probability weighted by gene-level LoF mutability in gnomAD (v.4.1) (Fig. 3c,d and Supplementary Table 20).

#### Enrichment for OCD common variant risk

Gene-level *z* scores were computed using MAGMA (v.1.10)^39^ from OCD GWAS^8^ meta-analysis summary statistics (23,493 cases and 1,114,613 controls of European ancestry, excluded 23andMe samples because of data sharing restrictions; https://pgc.unc.edu/for-researchers/download-results/), mapping SNPs to genes using GENCODE_v47 (snp-wise = mean) with 1000 Genomes European LD (Supplementary Fig. 12 and Supplementary Note 5).

#### Cross-disorder dnLGD overlap

We assessed whether genes affected by dnLGD were shared more than expected by chance among pairs of OCDCTD, ASD^26^, DD/ID^29^, SCZ^30^ and neurotypical SSC siblings (negative control). We focused on dnLGD because they were consistently reported across all WES studies. Observed gene overlap, null distributions, *z* scores and Benjamini–Hochberg-corrected *P* values were computed as above (Fig. 3e, Supplementary Fig. 13 and Supplementary Table 21). To quantify overlap magnitude, we additionally performed one-sided Fisher’s exact tests (background: 18,111 TADA-evaluated genes; Benjamini–Hochberg-corrected).

#### Comparison of gene-level association across disorders

The *q* values from recent WES studies of OCD and CTD (this study), ASD, NDD and SCZ^26,29,30^ were transformed as –log_10_(*q*), capped at 10^−8^, min–max-scaled within disorder and visualized using ternary plots (Supplementary Note 6, Supplementary Fig. 14 and Supplementary Table 22).

### Region-specific, age-specific and cell-specific expression of risk genes

#### GTEx

We assessed tissue-specific expression of hcOCDCTD genes across 54 Genotype Tissue Expression Project^41^ (GTEx v.10) tissues (bulk RNA-seq, log_10_(TPM + 1)). Tissue specificity was quantified by *t*-statistics comparing focal tissue expression against all others (excluding related tissues)^48^. Using the intersection of study and GTEx genes (*n* = 17,970) as background, we performed gene set enrichment analysis (fgsea v.1.24.0) by ranking genes by *t*-statistic and testing for enrichment using a one-sided model (scoreType = ‘pos’). *P* values were Benjamini–Hochberg-corrected (Fig. 4 and Supplementary Table 23).

#### BrainSpan and macaque

Temporal expression was evaluated using BrainSpan^42,43^ (microarray and RNA-seq; www.brainspan.org) in the neocortex, striatum, thalamus and cerebellum. Regions were selected for adequate sample size and analyses were conducted separately for each region. Samples were grouped by age: prenatal, infancy or childhood (0–12 years), adolescence (12–20 years) and adulthood (≥20 years). Significance was assessed by one-sided Wilcoxon rank-sum tests (background: TADA genes evaluated in BrainSpan; Benjamini–Hochberg-corrected; 60 comparisons). ASD genes served as a prenatal bias control^25,26^ and AD genes^49^ (from Bellenguez (2022) Supplementary Table 5) served as a postnatal bias control (Fig. 4, Supplementary Fig. 15 and Supplementary Table 24). We performed the same analysis with a rhesus macaque developmental brain transcriptome dataset^44^, which has lower variability in sample collection and postmortem quality (Fig. 4 and Supplementary Table 24).

#### Cell type enrichment

We evaluated enrichment of hc risk genes in mouse or human brain cell types using three independent datasets. In Zeisel (2018)^45^ (mouse nervous system scRNA-seq), we tested overlap with the top 10% most specific markers per cell type (from Bryois (2020)^50^ Supplementary Table 15) using one-sided Fisher’s exact tests (background: genes evaluated in both studies) (Fig. 4, Supplementary Fig. 16 and Supplementary Table 25). In Xu (2014) (mouse ^46,73^ data of CNS cell types), we tested overlap by FET using mouse brain cell type markers with specificity index *P* values < 0.05 (pSI and pSI.data R packages; https://sites.wustl.edu/doughertylab/psi_package-page/) mapped to human orthologs using Ensembl v.75 (Fig. 4, Supplementary Fig. 16 and Supplementary Table 26). Lastly, in Siletti (2023)^47^ (human brain snRNA-seq), we computed gene specificity as supercluster TPM/total TPM and evaluated enrichment using GSEA as with GTEx (Fig. 4, Supplementary Fig. 16 and Supplementary Table 27). All analyses were Benjamini–Hochberg-corrected for cell types and risk gene sets.

### Gene set enrichment analysis

Gene set overrepresentation analysis was performed in NetworkAnalyst^51^ with the union of hcOCDCTD and pOCDCTD genes for GO:BP and GO:MF terms (accessed June 9, 2025, default parameters, Benjamini–Hochberg correction; Supplementary Fig. 17 and Supplementary Table 28).

### Risk gene connectivity

We assessed the connectivity of hc and p gene sets in two networks: BioGRID v.4.4.241 (multivalidated physical PPIs) and PCNet v.2.2 (multimodal functional interactions, excluding cocitation edges, downloaded from www.ndexbio.org), with identifiers updated to current HGNC symbols^52,56^. We evaluated two connectivity metrics: (1) within-set connectivity, the number of risk genes directly connected to another in the set, with one-sided *P* values and *z* scores calculated from 10,000 size-matched permutations from the network background, and (2) interactome overlap, testing whether risk gene pairs shared more interactors than chance (one-sided Fisher’s exact per unique risk gene pair; background: intersection of TADA and network dataset genes), with empirical *P* values and *z* scores derived by comparing the percentage of significant pairs (*P* < 0.05) to a null from 1,000 samples of 100 random pairs (Fig. 5b,c, Supplementary Fig. 18 and Supplementary Tables 29 and 30).

### Rare and common variant convergence in interactomes

We tested whether BioGRID or PCNet first-degree interactors of hc seed genes (seeds excluded as interactors) were enriched for genes carrying dnLGD variants in trio probands or for OCD common variant genes (background: network-represented TADA genes). For rare variants, we compared the burden of dnLGD versus dnSyn variants (from trio probands) among interactor genes. For common variants, we tested interactors for enrichment of OCD GWAS-implicated genes (positional or functional). Both used two-sided Fisher’s exact tests with Bonferroni correction and 10,000-permutation empirical *P* values. Enrichment was considered significant if both the Bonferroni-adjusted Fisher *P* value and the empirical *P* value were <0.05 (Fig. 5d,e and Supplementary Tables 31,32). We additionally tested the association of OCD common variant risk^8^ on first-degree interactors using MAGMA gene set analysis^39^ (Supplementary Fig. 18 and Supplementary Table 33).

### Network propagation and functional enrichment

We evaluated the functional roles of hc risk genes using network propagation, which distributes signals from a set of seed risk genes across the PCNet (v.2.2) interaction network to quantify how functionally connected each gene is to the seed set. Analyses were performed separately for hcOCD (*n* = 12), hcCTD (*n* = 10) and the union of hcOCD, hcCTD and hcOCDCTD (*n* = 36) seeds. Using random walk with restart^88^ (restart probability 0.1; seeds = 1, others = 0), we computed gene-level empirical *P* values from 20,000 label permutations. The top 100 genes most strongly connected to each seed set underwent GO:BP (v.7.5.1) overrepresentation analysis (clusterProfiler::enricher (minGSSize = 10, maxGSSize = 500 (default), pAdjustMethod = ‘fdr’, universe = all PCNet genes in GO:BP, pvalueCutoff and qvalueCutoff = 1.1 to retain all terms for post hoc simplification and clustering). Redundant terms were clustered by Jaccard similarity, fully overlapping terms collapsed and the top ten representative terms per set according to lowest adjusted *P* value were visualized (Supplementary Fig. 19 and Supplementary Table 34).

### Comparison to structural variants

To contextualize our coding findings with structural variation, we curated CNVs from studies reporting elevated dn or rare CNV burden in OCD and CTDs^20,63–65^. This included OCD dnCNVs^64^ (*n* = 12), CTD dnCNVs^20^ (*n* = 36 from Supplementary Table 3, restricting to ‘cohort’ in ts-phase1, ts-phase2 or array and ‘confirmation status in this study’ reported as TRUE or Not_attempted), OCD rare CNVs^63^ (*n* = 494; Cochran–Mantel–Haenszel OR > 1) and CTD rare CNVs^65^ (*n* = 1,748 from Supplementary Table 4, restricting to individuals with Tourette syndrome (TS = 2) and retaining only genic CNVs (GENE_COUNT is not NA)). Coordinates were lifted from hg19 to GRCh38 and CNV-overlapping genes were intersected with hc genes, genes affected by dnDam, SFARI Tier 1S (ASD)^24^, Fu (2022) (ASD)^26^, Singh (2022) (SCZ)^30^ and genes implicated by OCD^8^ or SCZ^72^ GWAS. Enrichment was tested using one-sided hypergeometric tests (continuity-corrected ORs, Bonferroni-corrected) against the TADA background (Supplementary Tables 35 and 36).

### Use of generative AI

Portions of the methods and the main text were refined with assistance from ChatGPT (OpenAI, GPT-4o and GPT-5). All content was reviewed, edited and approved by the authors, who take full responsibility for the final paper.

### Reporting summary

Further information on research design is available in the Nature Portfolio Reporting Summary linked to this article.

## Online content

Any methods, additional references, Nature Portfolio reporting summaries, source data, extended data, supplementary information, acknowledgements, peer review information; details of author contributions and competing interests; and statements of data and code availability are available at https://doi.org/10.1038/s41593-026-02419-5.

## Supplementary information

Supplementary Information Supplementary Figs. 1–19, Table Legends, Notes 1–7 and References.

Reporting Summary

Peer Review File

Supplementary Tables 1–36 Supplementary Tables 1–36.

## Source data

Source Data Fig. 1 Cohort counts and statistical source data.

Source Data Fig. 2 Statistical source data, gene sets.

Source Data Fig. 3 Statistical source data.

Source Data Fig. 4 Statistical source data.

Source Data Fig. 5 Statistical source data.

## Data Availability

Sample metadata are provided in Supplementary Tables 2 and 3, dn mutations detected in trios are provided in Supplementary Table 6 and the summary statistics used as input for the TADA analysis, together with the TADA results, are provided in Supplementary Table 16. WES data newly generated in this study are available through the following repositories: TIC Genetics (phase 3; dbGaP phs001423.v4.p2, NDA collection 2958, NRGR studies 106 and 203) and FFOR (NDA collection 6223, NRGR study 227). This study also analyzed previously published WES data from the following cohorts, available under their original accessions: TIC Genetics Phase 1 (ref. ^21^) and Phase 2 (ref. ^20^) (BioProject PRJNA384374), TSAICG (BioProject PRJNA384389), OCGAS (dbGaP phs000903.v1.p1) and SSC (https://doi.org/10.15154/1149697)^89^. This study also used the following publicly available datasets: GRCh38 reference genome and contamination estimation reference data from the GATK resource bundle (https://gatk.broadinstitute.org/hc/en-us/articles/360035890811-Resource-bundle), population allele frequencies and constraint metrics from gnomAD (v.4.1; https://gnomad.broadinstitute.org/downloads), 1000 Genomes phase 3 release (https://www.internationalgenome.org/data-portal/data-collection), GENCODE (v.47; https://www.gencodegenes.org), MPC (v.2; https://github.com/broadinstitute/regional_missense_constraint/tree/main; based on gnomAD v.2.1.1), Polyphen-2 HDIV (v.2.2.3; accessed through Ensembl VEP v113), GTEx (v.10; https://gtexportal.org/home/downloads/adult-gtex/bulk_tissue_expression), BrainSpan (https://www.brainspan.org/static/download.html), the rhesus macaque developmental transcriptome (NCBI BioProject PRJNA448973), Zeisel (2018)^45^ mouse nervous system scRNA-seq cell type markers (Bryois (2020)^50^; Supplementary Table 15), Xu (2014) mouse BAC–TRAP cell type markers (pSI and pSI.data R packages; https://sites.wustl.edu/doughertylab/psi_package-page/), Siletti (2023) human brain snRNA-seq (adult_human_20221007.agg.loom; https://github.com/linnarsson-lab/adult-human-brain), SFARI Gene (Tier 1S, release January 13, 2025; https://gene.sfari.org), OCD GWAS summary statistics (https://pgc.unc.edu/for-researchers/download-results/), PCNet (v.2.2; https://www.ndexbio.org/search/?q=PCNet) and BioGRID (v.4.4.241; https://downloads.thebiogrid.org/BioGRID). Source data are provided with this paper.

## Code availability

All software tools used in this study are publicly available and described throughout Methods; a complete list with version numbers is provided in the Nature Portfolio Reporting Summary. Sequencing data were processed using GATK (v.4.2.0) with Picard (v.2.25.0), BWA-MEM (v.0.7.15-r1140) and VerifyBamID2 (v.1.0.6). Sample and variant quality control and analyses used Hail (v.0.2). Statistical analyses and visualization used R (v.4.2.3). Gene discovery used the TADA framework (https://github.com/talkowski-lab/TADA_2022). Custom code used for the TADA gene discovery analyses is available on GitHub (https://github.com/BelindaWangLab/OCDCTDphase1) and archived on Zenodo (https://doi.org/10.5281/zenodo.20804979)^90^.
