# Supplementary material for: Whole-exome sequencing in individuals with obsessive–compulsive disorder and chronic tic disorders identifies 36 large-effect risk genes
Source: Nat Neurosci. 2026 Sep 1;29(11):2659–71. doi: 10.1038/s41593-026-02419-5 (PMC13724258; doi:10.1038/s41593-026-02419-5)
Supplement: Supplementary file 2 [file 41593_2026_2419_MOESM2_ESM.pdf]

nature portfolio

Corresponding author(s): Matthew W. State, A. Jeremy Willsey, Thomas V. Fernandez, Gary A. Heiman, A. Moses Lee, Jay A. Tischfield

Last updated by author(s): Jun 22, 2026

Reporting Summary

Nature Portfolio wishes to improve the reproducibility of the work that we publish. This form provides structure for consistency and transparency in reporting. For further information on Nature Portfolio policies, see our Editorial Policies and the Editorial Policy Checklist.

Statistics

For all statistical analyses, confirm that the following items are present in the figure legend, table legend, main text, or Methods section.

n/a	Confirmed	
☐	☒	The exact sample size (*n*) for each experimental group/condition, given as a discrete number and unit of measurement
☐	☒	A statement on whether measurements were taken from distinct samples or whether the same sample was measured repeatedly
☐	☒	The statistical test(s) used AND whether they are one- or two-sided<br>*Only common tests should be described solely by name; describe more complex techniques in the Methods section.*
☐	☒	A description of all covariates tested
☐	☒	A description of any assumptions or corrections, such as tests of normality and adjustment for multiple comparisons
☐	☒	A full description of the statistical parameters including central tendency (e.g. means) or other basic estimates (e.g. regression coefficient) AND variation (e.g. standard deviation) or associated estimates of uncertainty (e.g. confidence intervals)
☐	☒	For null hypothesis testing, the test statistic (e.g. F, t, r) with confidence intervals, effect sizes, degrees of freedom and P value noted<br>*Give P values as exact values whenever suitable.*
☐	☒	For Bayesian analysis, information on the choice of priors and Markov chain Monte Carlo settings
☒	☐	For hierarchical and complex designs, identification of the appropriate level for tests and full reporting of outcomes
☒	☐	Estimates of effect sizes (e.g. Cohen's d, Pearson's r), indicating how they were calculated

Our web collection on statistics for biologists contains articles on many of the points above.

Software and code

Policy information about availability of computer code

Data collection	No software was used for data collection.
Data analysis	This is fully described in the Online Methods and Supplementary Information. In brief: sequencing data were processed using GATK_v4.2.0 and Picard_v2.25.0, with BWA-MEM_v0.7.15-r1140 for alignment to GRCh38 and VerifyBamID2_v1.0.6 (FREEMIX) for contamination estimation. De novo variants were scored with DeNovoCNN_v1.0 and manually reviewed in IGV_v1.14.0. Variants were annotated using the Ensembl VEP_v113 with the GENCODE_v47 transcript model. Sample and variant quality control were performed using Hail_v0.2 (https://hail.is/), with ancestry inference using scikit-learn_v1.3.1. Gene discovery used the TADA framework (https://github.com/talkowski-lab/TADA_2022). Gene-level common variant association used MAGMA_v1.10 (https://cncr.nl/research/magma/). Gene Ontology overrepresentation analysis used NetworkAnalyst (accessed 2025-06-09; https://www.networkanalyst.ca). The code used to generate the TADA gene discovery analyses is publicly available on GitHub (https://github.com/BelindaWangLab/OCDCTDphase1) and archived on Zenodo (DOI 10.5281/zenodo.20804914)<br><br>Statistical analyses and visualization were executed in R 4.2.3 using the following packages: AnnotationDbi 1.60.2, Biobase 2.58.0, BiocGenerics 0.44.0, biomaRt 2.54.1, broom 1.0.4, ComplexHeatmap 2.15.4, data.table 1.14.8, dplyr 1.1.2, eulerr 7.0.0, fgsea 1.26.0, forcats 1.0.0, GenomicRanges 1.50.2, ggplot2 3.5.2, ggpubr 0.6.0, ggrepel 0.9.3, ggtern 3.5.0, IRanges 2.32.0, limma 3.54.2, org.Mm.eg.db 3.16.0, paletteer 1.6.0, patchwork 1.3.0, pheatmap 1.0.12, pSI 1.1, pSI.data 1.0, purrr 1.0.1, readr 2.1.4, readxl 1.4.2, S4Vectors 0.36.2, Seurat 4.3.0.1, SeuratObject 4.1.3, sp 2.0-0, stringr 1.5.0, tibble 3.2.1, tidyr 1.3.0, tidyverse 2.0.0.

For manuscripts utilizing custom algorithms or software that are central to the research but not yet described in published literature, software must be made available to editors and reviewers. We strongly encourage code deposition in a community repository (e.g. GitHub). See the Nature Portfolio guidelines for submitting code & software for further information.

Data

Policy information about availability of data

All manuscripts must include a data availability statement. This statement should provide the following information, where applicable:

- Accession codes, unique identifiers, or web links for publicly available datasets
- A description of any restrictions on data availability
- For clinical datasets or third party data, please ensure that the statement adheres to our policy

Sample metadata are provided in Supplementary Tables 2 and 3; de novo mutations detected in trios in Supplementary Table 6; and the summary statistics used as input for the TADA analysis, together with the TADA results, in Supplementary Table 16. WES data newly generated in this study are available through the following repositories: TIC Genetics (Phase 3; dbGaP phs001423.v4.p2, NDA Collection #2958, NRGR studies #106 and #203) and FFOR (NRGR study #227). This study also analyzed previously published WES data from the following cohorts, available under their original accessions: TIC Genetics Phases 120 and 221 (BioProject PRJNA384374), TSAICG (BioProject PRJNA384389), OCGAS (dbGaP phs000903.v1.p1), and SSC (DOI 10.15154/114969793).

This study also used the following publicly available datasets: GRCh38 reference genome and contamination-estimation reference data from the GATK resource bundle (https://gatk.broadinstitute.org/hc/en-us/articles/360035890811-Resource-bundle); population allele frequencies and constraint metrics from gnomAD_v4.1 (https://gnomad.broadinstitute.org/downloads); 1000 Genomes phase 3 release (https://www.internationalgenome.org/data-portal/data-collection); GENCODE_v47 (https://www.gencodegenes.org); MPC_v2 (https://github.com/broadinstitute/regional_missense_constraint/tree/main; based on gnomAD_v2.1.1), Polyphen-2 HDIV_v2.2.3 (accessed through Ensembl VEP v113), GTEx_v10 (https://gtexportal.org/home/downloads/adult-gtex/bulk_tissue_expression); BrainSpan (https://www.brainspan.org/static/download.html); the rhesus macaque developmental transcriptome (NCBI BioProject PRJNA448973); Zeisel 2018 mouse nervous system scRNA-seq cell type markers (Bryois et al 2020 Supplementary Table 15); Xu 2014 mouse BAC-TRAP cell typemarkers (pSI and pSI.data R packages, https://sites.wustl.edu/doughertylab/psi_package-page/); Siletti 2023 human brain snRNA-seq (adult_human_20221007.agg.loom, https://github.com/linnarsson-lab/adult-human-brain); SFARI Gene (Tier 1S, release 2025-01-13; https://gene.sfari.org); OCD GWAS summary statistics (https://pgc.unc.edu/for-researchers/download-results/); PCNet_v2.2 (https://www.ndexbio.org/search/?q=PCNet); and BioGRID_v4.4.241 (https://downloads.thebiogrid.org/BioGRID).

Research involving human participants, their data, or biological material

Policy information about studies with human participants or human data. See also policy information about sex, gender (identity/presentation), and sexual orientation and race, ethnicity and racism.

Reporting on sex and gender	Both male and female participants were included in this study. Sex was inferred genetically. Primary analyses were not stratified by sex due to limited sample sizes, but sex was included as a covariate in singleton analyses. We conducted an exploratory analysis to evaluate whether sex was associated with different rates of de novo damaging variants.
Reporting on race, ethnicity, or other socially relevant groupings	Genetic ancestry was computationally inferred. For singleton data, to account for population stratification, only European samples were included.
Population characteristics	The study included 2,418 parent–proband trios in which the proband had OCD and/or CTD (7,254 individuals), 1,546 OCD singletons, and 1,734 neurotypical control trios (5,202 individuals). Both sexes were included; genetically inferred sex and ancestry (reported in Supplementary Tables 2–3) were used as covariates in singleton burden analyses, which were restricted to individuals of European ancestry. Age at onset was available for a subset of probands (Supplementary Table 2); age at assessment was not uniformly available across the aggregated cohorts and was not used as a covariate.
Recruitment	Subjects were recruited through the Tourette International Collaborative Genetics (TIC Genetics) and other cohort studies, or acquired through existing biorepositories. Additional case and control data were aggregated from previous publications. Ascertainment criteria and publications that describe ascertain strategies of each collection are provided in Supplementary Note 1 and Supplementary Table 1.
Ethics oversight	The Institutional Review Board of every site approved the study. All participants provided informed consent for genetic analyses (or parental consent for minors). Where provided, participant compensation consisted of a gift card up to $50. Only data from those who consented to share their genetic data through approved repositories have been deposited.

Note that full information on the approval of the study protocol must also be provided in the manuscript.

Field-specific reporting

Please select the one below that is the best fit for your research. If you are not sure, read the appropriate sections before making your selection.

☒ Life sciences ☐ Behavioural & social sciences ☐ Ecological, evolutionary & environmental sciences

For a reference copy of the document with all sections, see nature.com/documents/nr-reporting-summary-flat.pdf

Life sciences study design

All studies must disclose on these points even when the disclosure is negative.

Sample size	Sample sizes for all experiments in this study reflected the maximum achievable given available funding, recruitment efforts, and access to external data at the time of analysis. For gene discovery analyses, we included all sequenced OCD, CTD, and neurotypical control samples, restricting to families in which all members passed stringent quality-control criteria. For singleton analyses, we included all samples mapping to European ancestry.
Data exclusions	Samples were excluded from all analyses if they demonstrated evidence of poor sequence quality, based on coverage, read duplication, alignment rate, and PCR contamination metrics, or if they showed unexpected duplication or relatedness with other samples. For trio analyses, families were excluded if relatedness patterns were inconsistent with the expected family structure (unrelated parents, mother–child and father–child relatedness), the trio did not have one male and one female parent, or if the proband had an outlier (excess) of de novo mutations. We additionally excluded case trios in which a parent was affected (OCD or CTD). For singleton analyses, we retained only individuals genetically inferred to be of European ancestry to limit population stratification, and removed sample outliers based on call rate, singleton count, and heterozygous/homozygous and transition/transversion ratios. Exclusion criteria were established prior to burden testing with damaging variants.
Replication	We compared rates of de novo LGD, de novo Mis3, and rare LGD variants reported in prior studies with rates in our full dataset, as well as in subsets of previously published samples, and observed consistent results (PMIDs 30257206, 34183866). Previously reported de novo variants were replicated in our analysis of the same samples (PMIDs 31771860, 34183866, 28472652, 30257206). Because our study included many previously reported samples, we did not attempt an independent replication of published results. Nonetheless, our findings do not alter the designation of hcOCD genes CHD8 and SCUBE1 or hcCTD genes CELSR3 and WWC1. We additionally identified a new dnMis3 variant in WWC1 from a proband not included in prior CTD-focused studies. Comparisons with previously reported OCD and CTD risk genes are provided in Supplementary Note 4.
Randomization	Randomization is not applicable to the current study.This study utilized naturally occurring genetic variation as the experimental condition. Because an individual's genotype is fixed at conception, we could not randomly assign participants to experimental groups. Instead, we examined the association between existing genetic profiles and the observed phenotypes of OCD and/or CTD.
Blinding	Blinding is not relevant to the current study. Samples were not allocated to different conditions by the researches, and the phenotype ascertainment process is fully separate from the genotyping process.

Reporting for specific materials, systems and methods

We require information from authors about some types of materials, experimental systems and methods used in many studies. Here, indicate whether each material, system or method listed is relevant to your study. If you are not sure if a list item applies to your research, read the appropriate section before selecting a response.

Materials & experimental systems

n/a	Involved in the study
☒	☐ Antibodies
☒	☐ Eukaryotic cell lines
☒	☐ Palaeontology and archaeology
☒	☐ Animals and other organisms
☒	☐ Clinical data
☒	☐ Dual use research of concern
☒	☐ Plants

Methods

n/a	Involved in the study
☒	☐ ChIP-seq
☒	☐ Flow cytometry
☒	☐ MRI-based neuroimaging

Plants

Seed stocks	*Report on the source of all seed stocks or other plant material used. If applicable, state the seed stock centre and catalogue number. If plant specimens were collected from the field, describe the collection location, date and sampling procedures.*
Novel plant genotypes	*Describe the methods by which all novel plant genotypes were produced. This includes those generated by transgenic approaches, gene editing, chemical/radiation-based mutagenesis and hybridization. For transgenic lines, describe the transformation method, the number of independent lines analyzed and the generation upon which experiments were performed. For gene-edited lines, describe the editor used, the endogenous sequence targeted for editing, the targeting guide RNA sequence (if applicable) and how the editor was applied.*
Authentication	*Describe any authentication procedures for each seed stock used or novel genotype generated. Describe any experiments used to assess the effect of a mutation and, where applicable, how potential secondary effects (e.g. second site T-DNA insertions, mosiacism, off-target gene editing) were examined.*
