# Supplementary material for: Whole-exome sequencing in individuals with obsessive–compulsive disorder and chronic tic disorders identifies 36 large-effect risk genes
Source: Nat Neurosci. 2026 Sep 1;29(11):2659–71. doi: 10.1038/s41593-026-02419-5 (PMC13724258; doi:10.1038/s41593-026-02419-5)
Supplement: Supplementary file 3 [file 41593_2026_2419_MOESM3_ESM.pdf]

# Peer Review File

# Whole exome sequencing in individuals with obsessive-compulsive disorder and chronic tic disorders identifies 36 large-effect risk genes

Corresponding Author: Dr Matthew State

Version 0:

Reviewer comments:

Reviewer #1

(Comments for the Author)
The authors report a sequencing analysis of ~4K individuals, split between trios and singletons, with either obsessive compulsive disorder (OCD) chronic tic disorder (OCD) or both. Consistent with smaller earlier studies, the authors show a significant excess of de novo and rare damaging mutations in the cases compared with controls and a set of 36 high confidence genes with an excess of such variants. The hc genes overlap between OCD and CTD and with those found in other neuropsychiatric diseases but not heart disease. This is the strongest demonstration to date of the role of high impact rare variation in these disorders and a welcome, although unsurprising, conclusion. The methodology is convincing and I have no concerns about the conclusion. One suggestion for the authors to consider is an analysis of whether the clinical presentation differs for those that do and do not carry implicated high impacted rare variants. I would not make acceptance contingent on this, but if the data were available, this wouidl certainly make the study more interesting.

(Remarks on code availability)

Reviewer #2

(Comments for the Author)
This paper summarizes a large-scale analysis of individuals with OCD and/or CTD using exome sequencing and advanced statistical analysis. The research is conducted by a team that understands the phenotype, design and collection of samples, and the methods of analysis. Their interpretation of the results is sophisticated yet conservative in interpretation. My comments are largely constructive in nature, the paper could be published as is (significant time must have been taken to write the main text, display items, and methods/supplemental). Commets are presented in order of appearance in the text:
-Title: "risk" may still be fine to use in OCD/CTD but I wonder if "susceptibility" would be better given the move for a softer language to be used in psychiatric conditions, at least in the title and possibly abstract
-Abstract: the Abstract could have much more impact by listing the actual top genes of the 36 found and in particular the four overlapping the GWAS loci.
-Introduction: Heritability estimates are mentioned with proper references provided but I would also suggest listing the actual heritability numbers in the text so the reader can get better background of what the results may find (based on other rare variant studies say in autism and schizophrenia). I think it would be also useful in the Introduction to list the sex bias in OCD/CTD if one exists.
-Results: the 'control' trios used were the unaffected siblings of individuals with autism from the Simons Simplex Collection. Was the sequencing to generate this data performed on the same technology and exon capture array platform and what might any variances have on the data presented. I am not so worried about the sibs as controls but the comparability of the data given likely differences in the years this data was collected. I may have missed it but where was the exome sequencing done for this project...one site, multiple sites?
-variant calling seems to be of high-quality, but it seems no attempt was made to call CNVs (large or small), which should be assessable even via the exome data? Goes back to my earlier question, was there a reason for this. I would be interested to know if genes like NRXN1 (deletions mostly found in autism), deletions of ASTN2 (OCD), MBD5 (duplications), or larger CNVs like 16p11.2 and 1q21.1...there should be comment and more thorough referencing of earlier papers looking at CNVs in OCD (perhaps a supplemental table...do any of the genes found in these studies overlap, or not, with the ones found in the current study). What about RNU4-2....other RNU's if this was not analyzed it should be commented on (I will bet you will find mutations).
-more recently in autism and cerebral palsy mitochondrial variants were found to contribute in a not so insignificant manner

in rare variant analyses. These were from whole genome sequencing studies (not exome). However, could you call mitochondrial variants from the exome sequence? Either way, for mitochondrial, CNVs and other structural variants, if the data is not included there should be a section in the Discussion of 'limitations'.
-Figure 3 is nicely done. The genetic findings are the main ones of this paper that should be featured in the Abstract, presented in this manner the paper will have more impact.
-Data seems to be submitted to the proper databases, thank you.
-Other than earlier comments, the paper is well-referenced and will be a valuable and impactful contribution to the literature.

(Remarks on code availability)

Version 1:

Reviewer comments:

Reviewer #1

(Comments for the Author)

(Remarks on code availability)

Reviewer #2

(Comments for the Author)
This is a high-quality manuscript and with the edits it is ready for the next steps towards publication.

(Remarks on code availability)

**Open Access** This Peer Review File is licensed under a Creative Commons Attribution 4.0 International License, which permits use, sharing, adaptation, distribution and reproduction in any medium or format, as long as you give appropriate credit to the original author(s) and the source, provide a link to the Creative Commons license, and indicate if changes were made.
In cases where reviewers are anonymous, credit should be given to 'Anonymous Referee' and the source.
The images or other third party material in this Peer Review File are included in the article's Creative Commons license, unless indicated otherwise in a credit line to the material. If material is not included in the article's Creative Commons license and your intended use is not permitted by statutory regulation or exceeds the permitted use, you will need to obtain permission directly from the copyright holder.
To view a copy of this license, visit https://creativecommons.org/licenses/by/4.0/

## Response to referees

We thank the editor for giving us the opportunity to revise our manuscript and the reviewers for their thoughtful, constructive feedback. We are pleased they felt that our manuscript represented "the strongest demonstration to date of the role of high-impact rare variation in these disorders" (R1) and "a valuable and impactful contribution" (R2). We have carefully reviewed all their comments and suggestions and addressed these in turn below. Overall, we have:

- Revised the abstract to more prominently highlight the genetic findings, adopting the recommendations of R2 and specifically naming high-confidence genes. Given space limitations that preclude listing all our highest confidence findings in the abstract, we have chosen to list those that replicate prior high-confidence associations and those that overlap across common and rare variant based gene discovery. This underscores one of only a few instances in the published literature where exome sequencing and GWAS results for a common neurodevelopmental syndrome have shown statistically significant overlap at the level of individual high confidence genes.
- More extensively described our approach to addressing the technical heterogeneity we confronted across cohorts and sequencing platforms, clarifying how we mitigated these challenges to ensure reliable results and how this study design, combining diverse technical resources, impacted the types of analyses that could be rigorously pursued, for example, limiting our ability to conduct CNV analyses from exome data.
- Added additional information regarding our approach to other types of rare variation, including CNVs, non-coding variants, and mitochondrial mutations. We now specifically call out limitations in this regard and have added supplementary analyses and tables addressing the overlap between our and other's prior CNV findings.

Our point-by-point responses to the reviewers' comments are provided below.

**Reviewer #1 (Comments for the Author):**

The authors report a sequencing analysis of ~4K individuals, split between trios and singletons, with either obsessive compulsive disorder (OCD) chronic tic disorder (OCD) or both. Consistent with smaller earlier studies, the authors show a significant excess of de novo and rare damaging mutations in the cases compared with controls and a set of 36 high confidence genes with an excess of such variants. The hc genes overlap between OCD and CTD and with those found in other neuropsychiatric diseases but not heart disease. This is the strongest demonstration to date of the role of high impact rare variation in these disorders and a welcome, although unsurprising, conclusion. The methodology is convincing and I have no concerns about the conclusion. One suggestion for the authors to consider is an analysis of whether the clinical presentation differs for those that do and do not carry implicated high impacted rare variants. I would not make acceptance contingent on this, but if the data were available, this wouidl certainly make the study more interesting.

We thank the reviewer for their assessment of the significance of our study and for the important suggestion to further assess clinical presentations. In addition to our existing analyses of genotype–phenotype correlations with respect to sex (male versus female; Supplementary Fig. 8), we have now updated Supplementary Fig. 8 and the manuscript to include new analyses examining (i) comorbidity

status (OCDwCTD versus OCDonly or CTDonly) and (ii) age at onset in relation to the presence of de novo damaging mutations. The latter analysis was motivated by prior evidence suggesting an increased yield of damaging variants among individuals with earlier onset.

As an initial step, we evaluated whether probands carrying de novo damaging mutations in any of the 36 hcOCDCTD genes differed in sex composition or age at onset compared to other probands and observed no significant differences. However, phenotypic data were available for only 37 of the 76 such probands, limiting statistical power for analyses restricted to high-confidence risk genes. Given this constraint, we next examined these phenotypes in relation to de novo damaging variants more broadly, allowing for a more adequately powered assessment; these analyses likewise yielded no significant associations (Supplementary Fig. 8).

Moreover, the reviewer's suggestion prompted us to revise the manuscript to include a more extensive discussion of our approach to assessing genotype-phenotype relationships. Specifically, we now clarify that our focus on rare coding variants, together with a study design that integrated multiple cohorts from different sources, including both newly recruited and existing samples, necessarily limited the scope of well-powered genotype-phenotype analyses.  In addition, we now note in the discussion that our rare variant gene discovery effort coincides with several large-scale consortia that are collecting more detailed and harmonized phenotypic data and assembling substantially larger case-control samples for common variant studies, which will afford greater opportunities for comprehensive genotype-phenotype investigation. Notably, all subjects included in the present study, along with associated genotyping data, have already been made available to two Tourette/OCD consortia, and all rare-variant whole-exome sequencing data generated here will likewise be made available immediately. We therefore anticipate that more extensive genotype–phenotype analyses will be forthcoming in the near future.

We have revised the results section (lines 239-244)
"Using Poisson regression, we observed no sex-specific differences in dn variant rates in any variant class (Methods; Supplementary Fig. 8; Supplementary Tables 11 and 12). We further examined relationships between dn variant status and additional clinical phenotypes, observing no differences by comorbidity (OCDwCTD versus OCDonly/unk or TDonly) or by age at onset (Methods; Supplementary Fig. 8). However, these genotype-phenotype analyses were constrained by relatively small subgroup sizes due to incomplete phenotypic data for many samples."

We have also updated the discussion section (lines 484-489):
"We explored selected genotype–phenotype relationships, including sex, comorbidity, and age at onset, and did not observe differences in dn variant rates or carrier status. However, these analyses were limited by sample size and incomplete phenotypic data from aggregation across multiple sources. Ongoing consortia efforts with larger, more deeply phenotyped cohorts will enable more comprehensive genotype–phenotype analyses."

**Reviewer #2 (Comments for the Author):**

This paper summarizes a large-scale analysis of individuals with OCD and/or CTD using exome sequencing and advanced statistical analysis. The research is conducted by a team that understands the phenotype, design and collection of samples, and the methods of analysis. Their interpretation of the results is sophisticated yet conservative in interpretation. My comments are largely constructive in

nature, the paper could be published as is (significant time must have been taken to write the main text, display items, and methods/supplemental). Comments are presented in order of appearance in the text:

-Title: "risk" may still be fine to use in OCD/CTD but I wonder if "susceptibility" would be better given the move for a softer language to be used in psychiatric conditions, at least in the title and possibly abstract

We are mindful of the potential for stigma in discussions of psychiatric genetics and the reviewer raises an important question regarding the wisdom of using the term “risk” instead of “susceptibility” as the latter tends to reflect a more nuanced approach to discussing the genetics of common psychiatric disorders. However, in this case, we chose to use the term risk deliberately based on two primary (related) considerations. First, we calculate, for each high-confidence gene, the odds ratio (OR), which very closely approximates the relative risk in the context of a rare outcome. In short, we are explicitly calculating “risk” (see, for example, Figure 2e). Moreover, we show that collectively, these mutations confer a median OR of 57, with our top genes showing ORs of up to ~210-fold, which again reflects a well-accepted statistical approach to estimate the risk ratio. We therefore felt that “risk” most accurately conveys both the nature of the statistical analyses and the comparatively large quantitative impact on disorder liability conferred by the rare mutations we identified (particularly in comparison to common alleles that confer one to two orders of magnitude less liability), while still explicitly recognizing that these variants are not fully penetrant.

-Abstract: the Abstract could have much more impact by listing the actual top genes of the 36 found and in particular the four overlapping the GWAS loci.

As noted above, while we agree with the comment on the value of listing specific genes, given the abstract's word limit, we prioritized a small subgroup. We chose to highlight high-confidence genes that replicate prior associations, as these represent robust genetic findings. Additionally, among our high-confidence genes, we found four that directly overlap with OCD GWAS loci, an unusual finding among common psychiatric and neurodevelopmental disorders. Consequently, we chose to highlight these as both high-confidence genes and as a potential point of convergence between common and rare variant “forms” of OCD and CTD.

The abstract now reads (lines 94-97):
“ We find an excess in cases of de novo and rare protein-damaging mutations and identify 36 hc genes (false discovery rate [FDR] < 0.1), including four previously identified hc genes (*CELSR3*, *CHD8*, *SCUBE1*, and *WWC1*) and four genes that overlap with OCD GWAS loci (*BRWD1*, *CELSR3*, *QRICH1*, *SYNE1*).”

-Introduction: Heritability estimates are mentioned with proper references provided but I would also suggest listing the actual heritability numbers in the text so the reader can get better background of what the results may find (based on other rare variant studies say in autism and schizophrenia). I think it would be also useful in the Introduction to list the sex bias in OCD/CTD if one exists.

We thank the reviewer for this suggestion. We revised the Introduction to include heritability estimates and sex differences in OCD and CTD.

The introduction now reads (lines 106-116):

"Both OCD and CTD are highly heritable, with heritability estimates of 50% for OCD and up to 77% for CTD…Clinically, OCD is characterized by intrusive thoughts or images (obsessions) and repetitive behaviors or mental acts (compulsions) performed to alleviate distress, typically beginning in late childhood or early adulthood, with a male predominance in childhood-onset cases[5,11,12]. CTD is defined by persistent, recurrent motor movements and/or vocalizations, often preceded by premonitory urges[6,7], with typical onset in early childhood, peak severity around 10-12 years, improvement during adolescence, and a strong male predominance[6,13]."

-Results: the 'control' trios used were the unaffected siblings of individuals with autism from the Simons Simplex Collection. Was the sequencing to generate this data performed on the same technology and exon capture array platform and what might any variances have on the data presented. I am not so worried about the sibs as controls but the comparability of the data given likely differences in the years this data was collected. I may have missed it but where was the exome sequencing done for this project...one site, multiple sites?

We thank the reviewer for raising this important point and agree that it was an omission in the initial submission. The issue of sample and technical heterogeneity is indeed critical to the design and execution of this study. Below, we summarize the strategies we used to address this heterogeneity and have updated the results, methods, and Supplementary Table 1 to more clearly describe both the sources of technical variation and the measures taken to mitigate them.

Because the CTD and OCD samples were collected across multiple sites and years, trios and singletons were characterized using four sequencing platforms and seven exome capture designs, whereas control trios from the Simons Simplex Collection (SSC) were sequenced using a single platform and capture. Only a subset of 327 case trios (and one singleton) were sequenced on the same platform as the controls. To address both the heterogeneity among cases and between cases and controls, we implemented several complementary strategies:

1. Focus on de novo variants: For all trios, parents and probands were sequenced on the same platform and capture design, providing matched internal controls and minimizing platform- and era-specific artifacts. Additionally, because de novo variant rates are comparable across ancestries, this design also reduces potential confounding due to population stratification.
2. Calibration of rare variant quality thresholds: For singleton rare variant analyses, where parental controls are unavailable and case and control platforms are largely non-overlapping, we empirically calibrated SNV quality thresholds (VQSLOD) for the exome capture designs best represented among singleton samples. We used trio data to determine appropriate quality cutoffs, focusing on synonymous variants, which are expected to be selectively neutral and therefore transmitted from parent to offspring at a 50:50 ratio. For each exome capture design, we selected the VQSLOD threshold that yielded the most balanced (50:50) transmission of synonymous variants, reasoning that deviation from this expectation primarily reflect technical calling bias rather than biological effects. These empirically determined, capture-specific thresholds were then applied to filter rare variants in the corresponding singleton case and control datasets.
3. Restriction to consensus callable intervals: For burden analyses, we restricted all tests to "consensus callable intervals," defined as genomic regions that were captured by all exome designs ("consensus") and sequenced at high depth and quality in ≥90% of both case and control samples ("callable"). Consensus callable intervals were defined separately for de novo

and rare variant analyses, and TADA parameters were estimated using burden results derived from these regions.

4. Empirical validation: Using these approaches, we observed no detectable differences in de novo or rare synonymous variant rates between cases and controls, supporting adequate calibration across datasets (see Fig. 1b-d and Supplementary Fig. 4). In addition, the observed magnitude of de novo and rare variant burden is consistent with prior OCD and CTD rare variant studies, including studies that used different control samples and analytical pipelines(Cappi et al. 2020; Halvorsen et al. 2021; Wang et al. 2018; Willsey et al. 2017).

We have now updated Supplementary Table 1 to include sequencing site(s), sequencing platform(s), and exome capture(s) for each sample source to improve transparency regarding potential sources of technical heterogeneity. We also revised the Results text to explicitly note this technical heterogeneity and to direct readers to the Methods, where these approaches are described in detail.

The updated results section now reads (lines 185-187):
"To reduce artifacts arising from technical heterogeneity in sequencing platform, coverage, and capture design across samples sequenced at different sites and years, analyses were restricted to coding regions meeting specified quality metrics across all sources (Supplementary Table 1, Methods)."

-variant calling seems to be of high-quality, but it seems no attempt was made to call CNVs (large or small), which should be assessable even via the exome data? Goes back to my earlier question, was there a reason for this. I would be interested to know if genes like NRXN1 (deletions mostly found in autism), deletions of ASTN2 (OCD), MBD5 (duplications), or larger CNVs like 16p11.2 and 1q21.1...there should be comment and more thorough referencing of earlier papers looking at CNVs in OCD (perhaps a supplemental table...do any of the genes found in these studies overlap, or not, with the ones found in the current study).

We thank the reviewer for this thoughtful comment regarding copy number variants (CNVs). In response, we revised the manuscript to more explicitly address CNVs in OCD and CTD by adding a Supplementary Note summarizing prior work on de novo and rare CNVs, clarifying why CNV calling was not performed in the present study, and including new supplementary analyses and tables to contextualize our findings relative to earlier CNV studies.

We agree that rare CNVs represent an important component of genetic risk for OCD and CTD, and that CNVs can, in principle, be inferred from exome sequencing data. However, we elected not to perform CNV calling in this study because, as discussed above, our multi-cohort design includes substantial heterogeneity in sequencing centers, sequencing technologies, and exome capture platforms across cases and controls. Such heterogeneity poses a particular challenge for CNV calling. Established exome-based methods such as GATK gCNV explicitly emphasize that "matching samples on exome probe hybridization kit design is critical… while other factors such as sequencing center and platform are also important" (Babadi et al. 2023, PMID 37604963). In our case, the limited availability of a platform-matched subset of case and control samples dramatically reduced statistical power. In addition, exome-based CNV calling lacks clear positive and negative control benchmarks analogous to those available for SNVs and small indels, limiting our ability to robustly assess CNV call quality across heterogeneous datasets.

To place our findings in the context of prior CNV studies in OCD and CTD, we expanded the manuscript in several ways. We added Supplementary Note 7: Additional sources of genetic variation in OCD and CTD, which reviews evidence for increased burden of rare and de novo CNVs in both disorders, highlights loci that have reached genome-wide significance in CTD (including *NRXN1* deletions and *CNTN6* duplications), and places these findings in the context of the current study.

We also added new Supplementary Tables 35 and 36, which compile previously reported de novo and rare CNVs associated with OCD and/or CTD and assess overlap with (i) high-confidence and probable risk genes identified here, (ii) genes affected by de novo or rare coding variants in our analyses, and (iii) genes implicated by OCD or schizophrenia GWAS and by prior ASD and schizophrenia sequencing studies. Formal enrichment analyses did not reveal significant global overlap, though several gene-level intersections were observed (e.g., *CHD8*, *ZMYM2*, *ULK1*). Associated methods can be found in lines 1377-1396.

Finally, we updated the discussion to clarify the scope of the present analyses (lines 494-500) : "These findings also underscore the importance of future studies investigating the full spectrum of genetic variation to clarify how different variant classes act independently and jointly to influence risk. In this context, the present analyses focus on rare coding SNVs and small indels (≤50 bp) and do not include other classes of rare variation, such as CNVs, noncoding variants, or mitochondrial DNA. A recent CTD CNV study using microarray genotyping data[77] included samples overlapping those analyzed here, and comparison of our findings with previously reported OCD and CTD CNVs[27,67,68,78] is detailed in Supplementary Note 7."

What about RNU4-2....other RNU's if this was not analyzed it should be commented on (I will bet you will find mutations).

We appreciate the Reviewer's comment regarding *RNU4-2* and other noncoding RNAs. In response, we revised the manuscript to clarify the scope and limitations of our analyses with respect to these noncoding RNAs

We agree that *RNU4-2* is of particular interest, as de novo variants in this noncoding small RNA have been causally linked to neurodevelopmental disorders. In our study, noncoding regions, including RNUs, were excluded from primary analyses due to inconsistent coverage. Following the Reviewer's suggestion, we examined our sequencing data for variants mapping to spliceosomal snRNAs (RNUs). Although some exome capture platforms provide partial coverage of these loci, *RNU* genes were not included in the consensus intervals shared across capture platforms, which defined the analyzable search space for calibrated case–control comparisons. Specific examination of *RNU4-2* showed sparse coverage, with no variants passing quality control thresholds in either trio or singleton case probands. We now note this limitation in Supplementary Note 7 and in the discussion as noted above (lines 494-500).

-more recently in autism and cerebral palsy mitochondrial variants were found to contribute in a not so insignificant manner in rare variant analyses. These were from whole genome sequencing studies (not exome). However, could you call mitochondrial variants from the exome sequence? Either way, for mitochondrial, CNVs and other structural variants, if the data is not included there should be a section in the Discussion of 'limitations'.

We thank the Reviewer for this thoughtful comment. In response, we revised the manuscript to more explicitly acknowledge these limitations throughout our Discussion rather than adding a standalone “Limitations” section, as we felt this allowed for more integrated contextualization of the findings; however, we defer to the Editor should a separate section be preferred.

We were unable to assess mtDNA variants because none of the exome capture panels used to generate case or control data included targeted coverage of the mitochondrial genome. The exclusion of CNVs, noncoding RNAs, and mitochondrial DNA is now noted in Supplementary Note 7 and in the discussion as noted above (lines 494-500).

-Figure 3 is nicely done. The genetic findings are the main ones of this paper that should be featured in the Abstract, presented in this manner the paper will have more impact.

We greatly appreciate the reviewer’s assessment of the usefulness of Figure 3. We have addressed their suggestion regarding highlighting specific genes above.

-Data seems to be submitted to the proper databases, thank you.

We thank the Reviewer for noting this.

-Other than earlier comments, the paper is well-referenced and will be a valuable and impactful contribution to the literature.

We thank both reviewers again for the very constructive suggestions and their overall assessment of the quality and significance of our work. As noted above, their comments highlighted multiple important topics that we have now addressed in more detail in the current revision, which we believe have substantially strengthened the manuscript.
